# Comparative phylogeography of endemic Azorean arthropods

**DOI:** 10.1186/s12862-015-0523-x

**Published:** 2015-11-11

**Authors:** Aristeidis Parmakelis, François Rigal, Thanos Mourikis, Katerina Balanika, Sofia Terzopoulou, Carla Rego, Isabel R. Amorim, Luís Crespo, Fernando Pereira, Kostas A. Triantis, Robert J. Whittaker, Paulo A. V. Borges

**Affiliations:** Department of Ecology and Taxonomy, Faculty of Biology, National and Kapodistrian University of Athens, GR15784 Athens, Greece; Azorean Biodiversity Group (CITA-A) and Platform for Ecological and Environmental Research (PEER), Universidade dos Açores, Departamento de Ciências Agrárias, Rua Capitão João d’Ávila, São Pedro, 9700-042 Angra do Heroismo, Terceira Portugal; CE3C – Centre for Ecology, Evolution and Environmental Changes/Azorean Biodiversity Group and Universidade dos Açores - Departamento de Ciências Agrárias, 9700-042 Angra do Heroísmo, Açores Portugal; Conservation Biogeography and Macroecology Programme, School of Geography and the Environment, University of Oxford, South Parks Road, Oxford, OX1 3QY UK; Center for Macroecology Evolution and Climate, Department of Biology, University of Copenhagen, Universitetsparken 15, DK-2100 Copenhagen, Denmark

**Keywords:** Araneae, Azores, Coleoptera, Colonization routes, Discrete phylogeography, Extinction, Hemiptera, Mitochondrial DNA

## Abstract

**Background:**

For a remote oceanic archipelago of up to 8 Myr age, the Azores have a comparatively low level of endemism. We present an analysis of phylogeographic patterns of endemic Azorean island arthropods aimed at testing patterns of diversification in relation to the ontogeny of the archipelago, in order to distinguish between alternative models of evolutionary dynamics on islands. We collected individuals of six species (representing Araneae, Hemiptera and Coleoptera) from 16 forest fragments from 7 islands. Using three mtDNA markers, we analysed the distribution of genetic diversity within and between islands, inferred the differentiation time-frames and investigated the inter-island migration routes and colonization patterns.

**Results:**

Each species exhibited very low levels of mtDNA divergence, both within and between islands. The two oldest islands were not strongly involved in the diffusion of genetic diversity within the archipelago. The most haplotype-rich islands varied according to species but the younger, central islands contributed the most to haplotype diversity. Colonization events both in concordance with and in contradiction to an inter-island progression rule were inferred, while a non-intuitive pattern of colonization from western to eastern islands was also inferred.

**Conclusions:**

The geological development of the Azores has followed a less tidy progression compared to classic hotspot archipelagos, and this is reflected in our findings. The study species appear to have been differentiating within the Azores for <2 Myr, a fraction of the apparent life span of the archipelago, which may indicate that extinction events linked to active volcanism have played an important role. Assuming that after each extinction event, colonization was initiated from a nearby island hosting derived haplotypes, the apparent age of species diversification in the archipelago would be moved closer to the present after each extinction–recolonization cycle. Exploiting these ideas, we propose a general model for future testing.

**Electronic supplementary material:**

The online version of this article (doi:10.1186/s12862-015-0523-x) contains supplementary material, which is available to authorized users.

## Background

Islands have long been used as natural testing grounds for the investigation of species dispersal, diversification and extinction [1]. Their well-defined geographical setting makes them especially suitable for the study of how natural selection, gene flow and genetic drift have combined to shape current biodiversity patterns [2, 3]. In addition, recognition of the importance of the geological dynamics of oceanic islands to the understanding of diversification patterns has been codified in the general dynamic model of oceanic island biogeography, which postulates a central role for island ontogeny (building, maturity and dismantling of islands) in patterns of endemism within remote archipelagos [4]. Studying genetic diversity of endemic island taxa may also provide invaluable insights into conservation issues [2, 5]. Due to genetic bottlenecks during colonization and lower subsequent population sizes [6], populations of insular species are also prone to be genetically impoverished, theoretically rendering them more sensitive to demographic perturbations, which in turn might impede the long-term persistence of the species [6, 7]. These and other similar generalizations about island species have, however, been challenged, for example, by studies that show that oceanic islands were the sources of colonization events to continental areas [8] or that insular populations do not always have low genetic diversity [9]. Therefore, there is a need to compile more data and empirical analyses on the geography of speciation and regional diversification on islands. Within this brief, comparative genetic assessments of island endemic species are of particular interest.

Located in the North Atlantic Ocean (37–40° N, 25–31° W), the Azores is one of the world’s most isolated archipelagos (i.e. 1400 km from the tip of the European continent and 1800 km east of North America). There are nine islands, aligned on a west/north-west to east/south-east axis (Additional file 1: Figure S1) and divided into three island groups: western (Corvo and Flores), central (Faial, Graciosa, Pico, São Jorge and Terceira), and eastern (Santa Maria and São Miguel). These islands are the result of an active volcanism associated with the divergence of the African, Eurasian and American tectonic plates. Lying over a 615 km-long axis, the distance between the western and central islands is 218 km, while the central and eastern island groups are 139 km apart. The maximum isotopic subaerial ages for the Azorean islands are reported to be between 8.12 and 0.25 million years (Myr), with Santa Maria being the oldest island and Pico the youngest [10–17]. Two recent publications have reported younger maximum ages for some of the islands (especially for Santa Maria) [18, 19] and while we recognize that there is uncertainty about the most appropriate ages to use, we regard the dates used herein as the best current estimates available for the maximum age of each island [9–16]. While the maximum age of the islands is a key parameter, it has to be noted that for half of the 8 Myr life-span of the archipelago, only one island, Santa Maria, was in existence and this island has experienced episodes of large-scale sector collapse interspersed with episodes of volcanic growth, such that much of the island is far younger than this maximum age, a pattern shared with other islands in the Azores (and elsewhere) [1, 5, 13–19]. Therefore, while the Azores have a total area of 2324 km^2^, it turns out that 62 % of the territory is very recent in origin, less than 1 Ma [20].

The Azores harbour a significant number of endemic species [21] and the fauna and flora of these islands have been the subject of intensive study over the last twenty-five years, both taxonomically [22] and ecologically/biogeographically [23–25]. However, genetic analyses of Azorean endemic lineages remain scarce and little is known of either the age of most lineages or the patterns of intra-archipelago colonization [26–28]. Moreover, the Azorean native forest (*laurisilva*), which almost entirely covered the islands before human settlement (*c.* AD 1440), has been reduced anthropogenically to around 2.5 % of its original area (<58 km^2^ in total) [29]. This has led several endemic, and particularly forest specialist species, to extinction or to the brink of extinction and must have greatly reduced population sizes of the majority of native forest arthropod species [30].

There has also been some debate about the overall level of endemism and of archipelagic radiations within the Azores, which are regarded as surprisingly low for such a remote oceanic archipelago. Hypotheses to explain this low level of endemism include: (a) the late Quaternary palaeoclimatic variation hypothesis, which posits that the climatic stability of the Azores during the Quaternary in contrast to the greater climatic variation of the remaining islands of Macaronesia, has had a negative effect on the Azorean inter-island allopatric speciation patterns [27], (b) the undetected or cryptic diversity hypothesis, according to which the low diversity of the Azores can largely be attributed to several genetically distinct lineages not having been detected yet as they remain hidden within morphologically similar forms [27, 28], (c) the intra-archipelagic missing stepping-stone and habitat diversity hypotheses, which posit that the Azores are too young, too small and too homogeneous to have hosted many *in situ* diversification events [20, 31], and (d) the anthropogenic extinction hypothesis, which stresses the importance of unrecorded extinctions of many species in the oldest and most disturbed islands, in explaining the current species richness of the Azores [24]. Hence, analyses of the levels of genetic diversity and population connectivity of Azorean endemic species are of considerable potential interest.

Herein, using a phylogenetic and population genetics framework built on three mtDNA markers (COI, 16S rRNA, 12S rRNA), we study the phylogeographic and genetic diversity distribution patterns exhibited by six widespread Azorean endemic arthropods, representing three of the major taxonomic groups (Araneae, Hemiptera and Coleoptera) of the Azorean native forest fauna. The species selected differ in some important fundamental biological attributes, such as dispersal abilities, habitat specialization (highly dispersive canopy vs. more sedentary epigean species) and finally, regional rarity. Through the analyses performed we: i) estimated the time frame of the colonization of the archipelago for each species, ii) inferred the inter-island colonization patterns for each species, iii) documented the inter-island levels of gene flow for each species, and iv) identified the species and islands exhibiting the highest levels of genetic diversity and correlated the inferred patterns with the biological and biogeographic attributes of the species and islands, respectively. Our findings enabled us to test the following predictions: (1) the levels of mtDNA sequence divergence exhibited in each species is proportional to the geological age of the archipelago, (2) the oldest islands of the archipelago harbour more genetic diversity compared to the more recently emerged, (3) the less dispersive (epigean) species exhibit higher levels of inter- and intra-island mtDNA sequence diversity, and (4) the inter-island colonization patterns, ancestral areas and differentiation time-estimate of each species are in concert with the geological evolution of the Azores. Finally, we discuss existing island biogeographical models with reference to the Azores and tentatively propose a novel general framework for the evolutionary history of Azorean endemic arthropod species.

## Results

### mtDNA sequence data

In total we generated 546, 441 and 395 sequences of COI, 16S rRNA and 12S rRNA, respectively, from the Azorean species (see Methods, Table 1, and for details per species, locus and fragment see Additional file 1: Table S1). The mean size of the COI fragments amplified from all studied species was 592 bp (526–732 bp) and the respective values for 16S rRNA and 12S rRNA were 593 bp (445–790) and 567 bp (492–642). In all species COI was the most variable marker. Based on this marker, the mean overall sequence divergence between conspecific individuals, as estimated by MEGA, was 1.6 % in *Gibarranea occidentalis*, 1.9 % in *Sancus acoreensis,* 0.3 % in *Savigniorrhipis acoreensis*, 2.6 % in *Aphrodes hamiltoni*, 1.9 % in *Pinalitus oromii* and 2.3 % in *Alestrus dolosus*. The respective values for the 12S rRNA fragment in all the species analysed ranged from 0.0 % (*Savigniorrhipis acorensis*) to 1.3 % (*A. hamiltoni*), with similar levels being shown by the 16S rRNA data (see Additional file 1: Table S4).

**Table 1:** Sequence data information

Species	mtDNA marker		
	COI	16S rRNA	12S rRNA
*Gibarranea occidentalis*	106	83	76
*Sancus acoreensis*	180	158	102
*Savigniorrhipis acoreensis*	76	76	76
*Aphrodes hamiltoni*	58	45	37
*Pinalitus oromii*	96	56	74
*Alestrus dolosus*	30	23	30
Total	546	441	395

The number of COI, 16S rRNA and 12S rRNA sequences generated for six endemic species of Azorean arthropods

### Population genetic analyses using COI

**Fig. 1:**
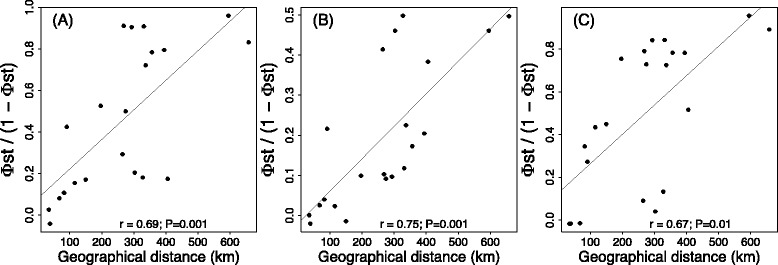
Correlations between pairwise linearized Φst and pairwise geographical distances (Mantel tests) to evaluate isolation by distance (IBD) in (**a**), *Gibarranea occidentalis* (**b**) *Sancus acoreensis*, and (**c**) *Pinalitus oromii* in the Azores. The Pearson's correlation coefficients (r) and P-values (*P*) for each test are provided in each panel

As the most variable marker in all species was COI, the population genetic analyses of all species relied solely on this marker. For these analyses we removed shorter sequences from the datasets, aiming to preserve as much information as possible during the haplotype inference analyses. After editing and trimming some of the COI sequences from each species, our datasets (aligned) included a 354 bp nucleotide matrix with 21 variable sites for *G. occidentalis*, with respective values for the other species being 420 bp with 82 sites for *Sancus acoreensis*, 427 bp with 5 sites for *Savigniorrhipis acoreensis*, 486 bp with 35 sites for *A. hamiltoni*, 480 bp with 58 sites for *P. oromii* and 455 bp with 35 sites for *A. dolosus.* Overall, diversity values (h, h_rar_, *Hd* and π) were consistently higher for *Sancus acoreensis* and *P. oromii* than for the other four species (Table 2)*.* Moreover, these two species display statistically significant negative values of Fu’s *F*_*S*_ for a substantial number of islands (4 out of 7 for both species), suggesting a past episode of demographic expansion for these populations (Pico, São Jorge, and Terceira islands for *Sancus acoreensis* and Faial, Flores, Pico and Terceira islands for *P. oromii*) (Table 2). Pairwise Φst analyses revealed significant divergence between islands for the three more genetically diverse species *G. occidentalis, Sancus acoreensis* and *P. oromii* (Additional file 1: Table S5). For *Sancus acoreensis*, only Pico, Faial and São Jorge were genetically distinct from each other. Terceira was not different from Faial but differed marginally, yet significantly, from Pico and São Jorge (Additional file 1: Table S5). Similarly, for *P. oromii* and *G. occidentalis,* Pico, Faial and São Jorge exhibited marginal genetic differences between each other. Moreover, for *G. occidentalis*, *Sancus acoreensis* and *P. oromii*, genetic divergence computed by Φst/(1- Φst) was highly and significantly correlated with geographical distance (Fig. 1)*.* However, the statistical parsimony networks revealed strongly contrasting patterns amongst these three species (Additional file 1: Figure S2). In *G. occidentalis*, both the central islands group and Flores were clearly differentiated from Santa Maria and São Miguel, with only one haplotype shared between Terceira and São Miguel (Additional file 1: Figure S2). Because of its extremely high haplotype diversity (0.989 ± 0.003), the network for *Sancus acoreensis* was not informative, showing mainly complex connections between most of the singleton and doubleton haplotypes (Additional file 1: Figure S2). Only Flores and São Miguel formed distinct haplotype groupings in the network. In *P. oromii*, Terceira and the eastern Azorean islands (Santa Maria and São Miguel) were clearly differentiated from each other, with a third group, encompassing the remaining central islands (Faial, Pico, São Jorge) together with Flores (Additional file 1: Figure S2).

**Table 2:** Genetic data analyses results

Species/sites	N	h	h_rar_	Uh	Hd (SD)	π (SD)	Tajima’s D	Fu’s F_S_
*Gibbaranea occidentalis*								
Archipelago	106	16			0.681 (0.043)	0.0089 (0.0010)	−0.11	0.36
Faial	18	3	1.250	1	0.542 (0.086)	0.0022 (0.0003)	0.78	1.38
Flores	16	2	0.313	1	0.125 (0.106)	0.0003 (0.0002)	−1.16	−0.7
Pico	16	6	1.514	2	0.742 (0.084)	0.0028 (0.0004)	−0.1	0.09
São Jorge	7	3	1.667	1	0.667 (0.160)	0.0022 (0.0008)	−0.65	0.11
Santa Maria	8	5	1.389	4	0.857 (0.108)	0.0113 (0.0018)	1.98	2.65
São Miguel	9	3	2.143	2	0.556 (0.165)	0.0021 (0.0007)	−0.36	0.35
Terceira	32	5	1.686	2	0.649 (0.066)	0.0095 (0.0014)	1.45	6.20
*Sancus acoreensis*								
Archipelago	159	115			0.989 (0.003)	0.0179 (0.0006)	−1.51*	−24.65***
Faial	5	5	4.000	4	1	0.0177 (0.0041)	−0.59	−0.73
Flores	26	14	2.904	14	0.858 (0.057)	0.0136 (0.0013)	−0.82	−2.55
Pico	32	26	3.826	21	0.982 (0.015)	0.0139 (0.0014)	−1.26	−18.28***
São Jorge	12	12	4.000	9	1	0.0121 (0.0017)	−1.19	−8.10***
Santa Maria	11	5	2.089	5	0.709 (0.137)	0.0087 (0.0025)	−1.02	1.06
São Miguel	24	21	3.860	18	0.986 (0.018)	0.0166 (0.0016)	−1.06	−12.31***
Terceira	49	43	3.903	38	0.990 (0.009)	0.0167 (0.0012)	−1.698	−25.12***
*Savigniorrhipis acoreensis*								
Archipelago	62	5			0.500 (0.057)	0.0026 (0.0003)	0.09	1.49
Faial	7	1	-	-	-	-	-	-
Flores	10	1	-	-	-	-	-	-
Pico	7	1	-	-	-	-	-	-
São Jorge	10	1	-	-	-	-	-	-
Santa Maria	5	2	-	2	0.400 (0.237)	0.0009 (0.0006)	−0.82	0.09
S.	6	1	-	-	-	-	-	-
Terceira	17	1	-	-	-	-	-	-
*Aphrodes hamiltoni*								
Archipelago	49	3			0.081 (0.053)	0.0055 (0.0036)	−2.32*	11.39
Faial	8	1	-	-	-	-	-	-
Flores	5	1	-	-	-	-	-	-
Pico	13	2	-	1	0.154 (0.126)	0.0146 (0.0120)	−2.36*	13.26
São Jorge	6	1	-	-	-	-	-	-
Santa Maria	3	1	-	-	-	-	-	-
Terceira	14	2	-	1	0.143 (0.119)	0.0096 (0.0096)	−2.40*	12.36
*Pinalitus oromii*								
Archipelago	80	49			0.969 (0.011)	0.0188 (0.0014)	−0.748	−24.62***
Faial	11	10	3.818	8	0.982 (0.046)	0.0460 (0.0460)	−1.35	−5.40**
Flores	12	5	1.667	4	0.576 (0.163)	0.0021 (0.0009)	−1.49*	−1.94*
Pico	11	9	3.636	5	0.964 (0.051)	0.0081 (0.0025)	−1.48	−3.64*
São Jorge	8	7	3.643	4	0.964 (0.077)	0.0130 (0.0036)	−0.79	−1.42
Santa Maria	6	5	3.333	5	0.933 (0.122)	0.0067 (0.0014)	0.25	−1.16
São Miguel	5	1	0.000	1	-	-	-	-
Terceira	27	20	3.728	18	0.972 (0.02)	0.0122 (0.0018)	−1.18	−9.67***
*Alestrus dolosus*								
Archipelago	25	10			0.823 (0.053)	0.0260 (0.0034)	1.05	5.93
Flores	5	1	-	1	-	-	-	-
Pico	3	1	-	1	-	-	-	-
São Miguel	12	3	-	3	0.439 (0.158)	0.0040 (0.0015)	−0.84	2.05
Terceira	5	3	-	3	0.700 (0.218)	0.0026 (0.0011)	−1.05	−0.19

Molecular diversity indices for six species of endemic arthopods in each of the Azorean islands investigated, based on COI sequence data. N, number of individuals; h, number of haplotypes; h_rar_, rarefied number of haplotypes; Uh, number of unique haplotypes; *Hd*, haplotype diversity; *π*, nucleotide diversity; SD, standard deviation. For Tajima’s D and Fu’s F_S_ * 0.01 < *P* < 0.05; ** 0.001 < *P* < 0.01 and *** *P* < 0.001 otherwise *P* > 0.05. The discordance between number of individuals (N) for COI between this table and Additional file 1: Table S1 is due to the fact that for this analysis individuals having shorter COI sequences were removed

### Phylogeographic analyses and estimate of divergence times

**Fig. 2:**
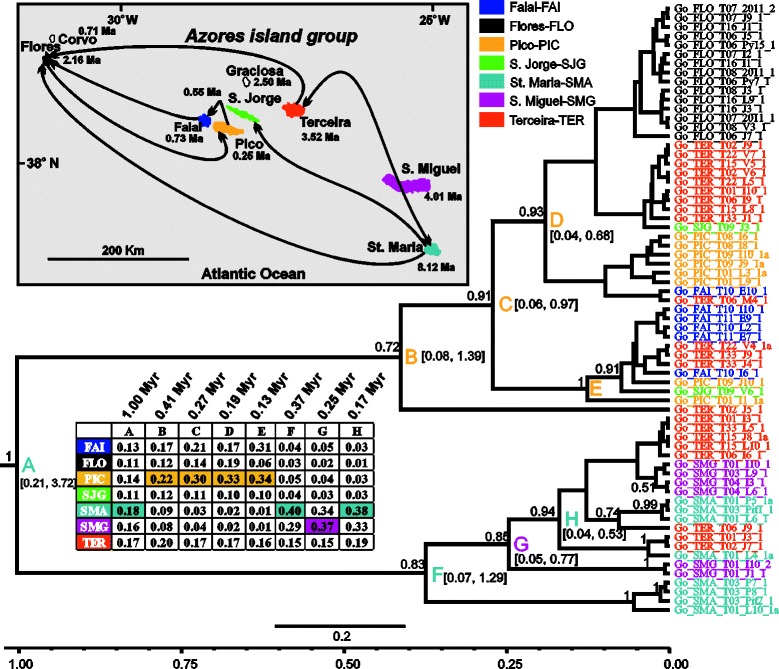
BEAST maximum clade credibility tree (concatenated 3 mtDNA genes dataset) of *Gibarranea occidentalis* showing the median age estimates of all the splitting events. Numbers on branches are the posterior probability values of the BEAST inference (only values above 0.5 are shown). The nodes of major splitting events are marked with coloured letters. The inset table presents the probability (state probability) of each colour-coded island (according to the legend) being the ancestral area of the respective letter-coded node. The median age corresponding to each letter-coded node is indicated in the table and the numbers within brackets are the 95 % HPD intervals. The scale bar and the time axis are in Ma. Tip names are colour-coded by location (island) of origin. The inset map depicts the current geographical setting of the Azores, black lines indicate strongly supported colonization events

**Fig. 3:**
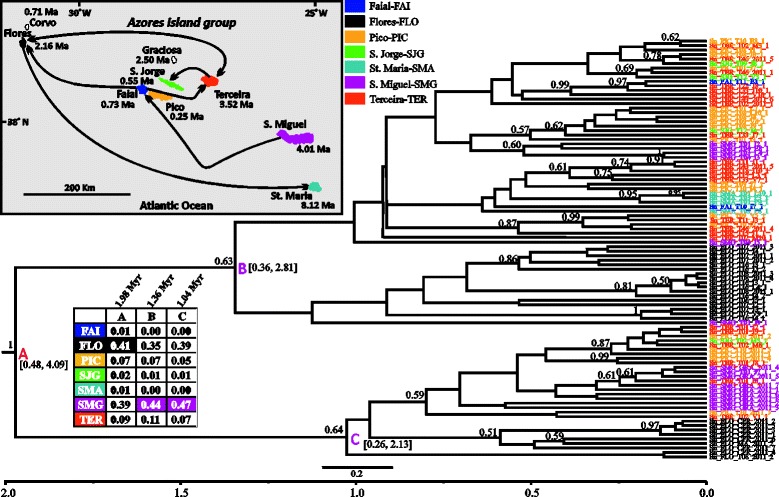
BEAST maximum clade credibility tree (concatenated 3 mtDNA genes dataset) of *Sancus acoreensis* showing the median age estimates of all the splitting events. Further details are explained in Fig. 2 (legend)

**Fig. 4:**
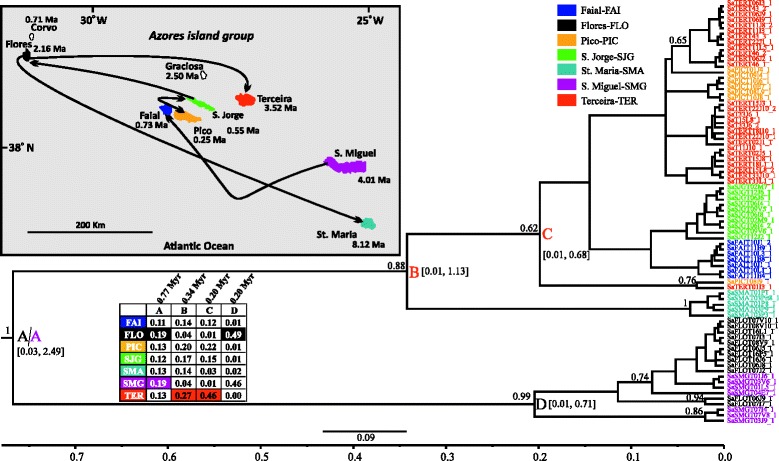
BEAST maximum clade credibility tree (concatenated 3 mtDNA genes dataset) of *Savigniorrhipis acoreensis* showing the median age estimates of all the splitting events. Further details are explained in Fig. 2 (legend)

**Fig. 5:**
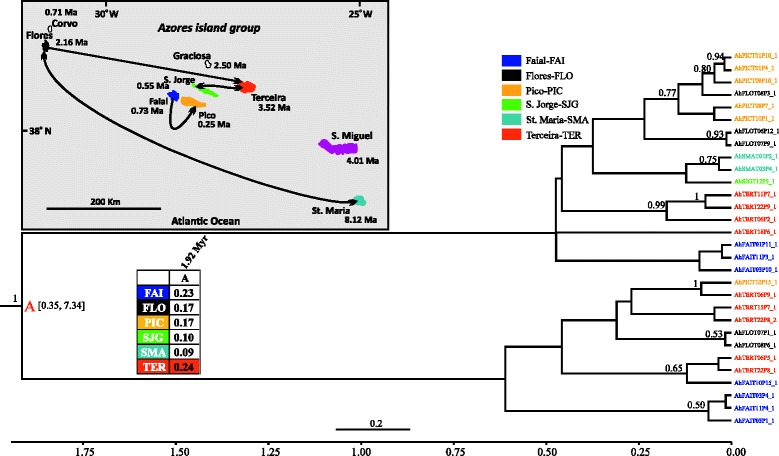
BEAST maximum clade credibility tree (concatenated 3 mtDNA genes dataset) of *Aphrodes hamiltoni* showing the median age estimates of all the splitting events. Further details are explained in Fig. 2 (legend)

**Fig. 6:**
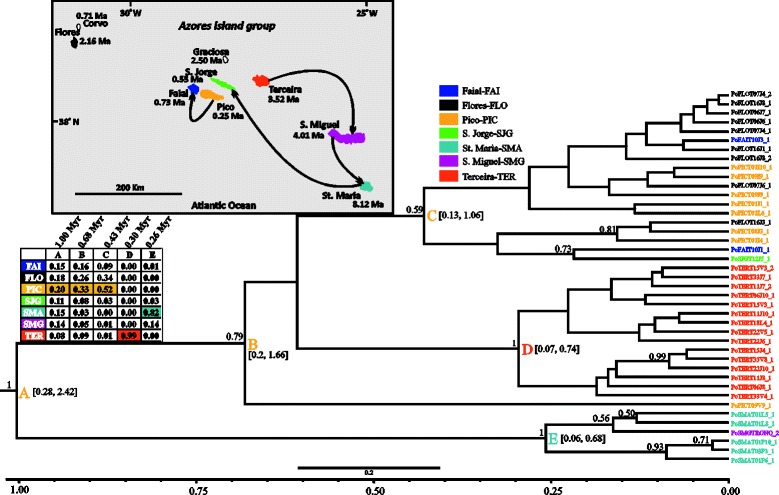
BEAST maximum clade credibility tree (concatenated 3 mtDNA genes dataset) of *Pinalitus oromii* showing the median age estimates of all the splitting events. Further details are explained in Fig. 2 (legend)

**Fig. 7:**
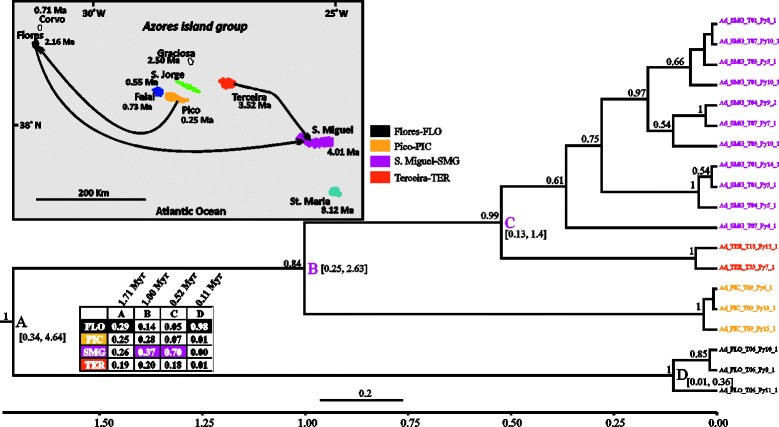
BEAST maximum clade credibility tree (concatenated 3 mtDNA genes dataset) of *Alestrus dolosus* showing the median age estimates of all the splitting events. Further details are explained in Fig. 2 (legend)

Detailed information regarding the datasets used in the phylogeographic analyses of each species is provided in Additional file 1: Table S6 and the results are presented in Figs. 2, 3, 4, 5, 6 and 7.

#### Gibarannea occidentalis

The first diversification event is dated at 1 Ma (node A: 0.21–3.72, 95 % HPD) and the majority of diversification events took place in the last 0.41 Myr (Fig. 2). Two well-supported major clades are recovered by the analysis. The first clade comprises specimens from Santa Maria, São Miguel and Terceira, whereas the second is composed of specimens from the remaining four islands (Flores, Faial, Pico, and São Jorge) and Terceira. Santa Maria (node A) is reconstructed as the most likely (state probability, sp: 0.18) ancestral range of the Azorean differentiation, but São Miguel (sp: 0.16) and Terceira (sp: 0.17) are almost equally plausible. A complex pattern of colonization events is inferred and most of them involve the western and central islands of the Azores. Individuals from Santa Maria colonized Terceira, São Jorge and Flores, and in turn Terceira lineages have colonized Flores. Santa Maria is best supported as the species’ ancestral colonization area and at least three colonization events have taken place from this island. No colonization events are supported to have occurred from São Miguel, whereas Terceira lineages seem to have colonized both Flores and Santa Maria.

#### Sancus acoreensis

This species has been differentiating within the Azores over the last 1.98 Myr (node A: 0.48–4.90, 95 % HPD), as evidenced by the first diversification event (Fig. 3). However, the remaining diversification events seem to have occurred within the last 1.12 Myr. The two major clades inferred by the analyses are weakly supported (posterior probability, 0.63 and 0.64) and both comprise specimens originating from almost all Azorean islands. Flores (node A, sp: 0.41) is reconstructed as the ancestral region of the species differentiation in the Azores, but is only marginally preferred to São Miguel (sp: 0.39). In the two major clades inferred, São Miguel (nodes B, C) is reconstructed as the most likely ancestral region, with sp values above 0.4. Flores, one of the islands supported as the starting point of the colonization, is the source of the colonization of Santa Maria. Lineages from São Miguel are supported as having colonized Faial. All the remaining colonization events involve the central and western islands of the Azores, with Flores having been colonized twice, once from Terceira and once from Faial. In addition, lineages from Flores seem to have colonized Terceira.

#### Savigniorrhipis acoreensis

The onset of the differentiation of this species is estimated at 0.77 Ma (0.44–2.49, 95 % HPD) and the majority of the remaining diversification events have taken place during the last 0.34 Myr (Fig. 4). Flores and São Miguel are equally likely to be the ancestral areas of the species differentiation within the Azores. Two major clades are strongly supported (nodes B and D) and these are further subdivided into subclades. One of the major clades (node B) hosts specimens originating from all Azorean islands except Flores. Terceira (node B: sp 0.27) is inferred as the ancestral region for this clade. The other major clade is composed only of specimens from Flores and São Miguel, with Flores (node D, sp: 0.49) being the ancestral region. Several colonization events are supported, with lineages from Flores having colonized Terceira and Santa Maria, and with individuals from São Miguel having colonized Faial. On the other hand, Flores has been colonized from São Jorge.

#### Aphrodes hamiltoni

For *A. hamiltoni* we have not succeeded in obtaining any sequences from samples collected from the island of São Miguel, and therefore this island is not included in the analyses. According to the tree (Fig. 5) it can be seen that the differentiation of the species within the archipelago was initiated around 1.92 Ma (node A: 0.36–5.40, 95 % HPD) from Terceira (node A, sp: 0.24). However, Faial (sp: 0.23), another central island, is also equally likely to be the starting point of the colonization. The tree is partly unresolved, thus fewer inferences regarding the colonization process can be made. Nevertheless, there is support for Flores having been colonized from Santa Maria and vice versa. Flores was also the colonization source of Terceira. The remaining events involve the central islands of the Azores.

#### Pinalitus oromii

This species is estimated to have been diversifying in the Azores for at least 1 Myr (Fig. 6: node A, 0.28–2.4, 95 % HPD), with the majority of diversification events occurring within the last 0.43 Myr. The island of Pico (node A, sp: 0.20) is reconstructed as the ancestral island of the species in the group, although Flores is also strongly supported (sp: 0.18). A few dispersal events are strongly supported and these involve the central and eastern Azorean islands. Pico has colonized Faial, whereas Terceira colonized Santa Maria. São Miguel is inferred as the source of the colonization of Santa Maria.

#### Alestrus dolosus

In *A. dolosus*, the time frame of differentiation in the Azores is estimated at 1.71 Ma (node A, 0.34–4.6, 95 % HPD). Most of the lineages diversified within the last 0.53 Myr. The inferred tree (Fig. 7) is very well resolved and several ancestral regions of descendent clades are reconstructed. Flores (node A, sp: 0.29) is the island inferred as the most likely ancestral area of the species differentiation in the archipelago, but both São Miguel and Pico have a high probability as well (0.26 and 0.25, respectively). São Miguel is the most frequently invoked ancestral island for several clades (nodes B, C, Fig. 7). However, the BSSVS analysis, which indicates putative colonization routes, failed to support any colonization events initiating from São Miguel. On the contrary, this island seems to have been colonized both from Terceira and Flores. Pico seems to have been the source of the colonization of Flores.

## Discussion

### Genetic diversity

All species analysed in this work are Azorean endemics with a widespread distribution in the archipelago. Each species has thus a history of diversifying solely within the Azores and therefore none of them host genetic diversity “imported” secondarily from external areas. All are native forest specialists but they possess different fundamental habitat adaptation attributes, namely occurring mostly in canopies of endemic trees (*Gibarranea occidentalis*, *Sancus acoreensis, Savigniorrhipis acoreensis*, *Pinalitus oromii*) or in the soil (*Aphrodes hamiltoni*, *Alestrus dolosus*). Considering (i) the geological ages of the oldest islands of the archipelago, (ii) the geographical extent of the Azores (615 km on an east–west axis) and (iii) the fact that the generally accepted rate of evolution of arthropod mtDNA is 2.3 % per Myr [32] or even higher [33], reasonable levels of divergence are expected between the mtDNA sequences of individuals originating from different sampling locations (populations). For this to be true some degree of isolation of the different populations should be in effect. Contrary to our predictions relating to the overall geological age of the archipelago, the levels of sequence divergence recorded in all species studied herein (mean overall pairwise divergence in the most variable mtDNA marker is between 0.3 and 2.6 %) are well below what is expected. Moreover, the observed low levels of genetic diversity do not seem to be specific to a particular arthropod group since we have included representatives of two different groups of arthropods, namely spiders and insects, with the insects being represented by two different orders, Hemiptera and Coleoptera. Interestingly, similar levels of low genetic diversity have been recorded in many other groups studied from the Azores, spanning from bats to bryophytes [8, 34–36]. Previous authors have generally posited recent colonization of the Azorean islands to explain the low levels of genetic diversity. Equally important, the results of our study do not provide evidence of any cryptic species within the six species considered herein, thus confirming their taxonomic integrity and multi-island Azorean endemic status [cf. 37].

The inferences drawn from the levels of sequence divergence can also be made from the analyses of the population genetics of the species (Table 2). Whereas the nucleotide diversity (*π*) of the haplotypes is low in all the species, regardless of the spatial level investigated (the whole archipelago or single islands), haplotype diversity (*Hd*) appears to be high in certain species (Table 2). On this basis we can discern two categories, species with (a) high overall and per-island *Hd* haplotypic diversity, namely *Sancus acoreensis*, and *P. oromii*; and species with (b) low overall and per-island *Hd,* namely *G. occidentalis*, *Savigniorrhipis acoreensis*, *A. hamiltoni* and *A. dolosus*. The islands with the highest *Hd* differ between the six species. In the case of *G. occidentalis*, Santa Maria has the highest *Hd* and hosts the highest number of unique haplotypes, with the remaining islands exhibiting lower values. For *Sancus acoreensis* it is mainly the central group of islands, namely Faial, Pico, São Jorge, and Terceira, together with the eastern island of São Miguel, that harbour most of the *Hd* and unique haplotypes, with Terceira being the richest. In *P. oromii* it is again the central group of islands, together with Santa Maria, which host most of the species haplotypes.

The inconsistency between the archipelago’s apparent geological age and levels of genetic diversity creates space for questioning the actual time that these taxa have been differentiating in the Azores. The low levels of genetic diversity they exhibit suggest either or both a much shorter diversification time on the Azores than assumed based on the archipelago’s ages reported in the literature or a dramatic loss of genetic diversity due to significant demographic changes. According to the neutrality tests it appears that for four species a departure from constant population size cannot be supported, whereas both the canopy spider *Sancus acoreensis* and the canopy bug *P. oromii* show signs of a recent population expansion (Table 2). Hence, notwithstanding the anthropogenic habitat loss of recent centuries, the signal for the latter two species is of recent diversification. The inference of geographical expansion within the archipelago is also corroborated by the complex and extended haplotype networks both species exhibit (Additional file 1: Figure S2). However, we cannot exclude the possibility of this recent expansion being preceded by cryptic past episodes of loss of genetic diversity.

### Divergence time estimates and colonization patterns

Based on the generated mtDNA sequence data we were able to infer a time-calibrated tree for each of the species studied (Figs. 2, 3, 4, 5, 6 and 7). Furthermore, by applying the CTMC model of discrete phylogeographic analysis [38] we reconstructed the ancestral locations (islands) of several nodes strongly or adequately supported in the phylogenetic trees of each species studied. In general, the reconstructed trees can be considered well-resolved based on posterior probability values of clades (*G. occidentalis* and *A. dolosus*), moderately resolved (*Savigniorrhipis* acoreensis), or unresolved (*Sancus acoreensis*, *A. hamiltoni* and *P. oromii*). For the latter cases, we should treat the ancestral range reconstruction, and the BSSVS analysis, with caution. Furthermore, it has to be noted that in the majority of the species analysed, sequences originating from the same island do not appear as monophyletic groups in the trees but are scattered throughout the trees. This is the reason for not imposing any time constraints during the inference of the time-calibrated trees.

According to the literature, the Azores date back to around 8 Ma, but for the first half of this period, only one island, Santa Maria, was in existence, and it was not a particularly large island. Moreover, Santa Maria has experienced considerable volcanic and land-surface disturbance, with large scale slippage events dumping significant parts of the island back into the sea at intervals [19]. Despite such destructive episodes, the number of islands and the total land area of the Azores have each increased considerably over time. Focusing on the divergence time estimates, all species appear to have started differentiating in the Azores within a timespan ranging between 0.77 (95 % HPD intervals: 0.03–2.49, *Savigniorrhipis acoreensis*) and 1.98 Ma (95 % HPD intervals: 0.48–4.09, *Sancus acoreensis*) and, in all species except *Sancus acoreensis*, the majority of diversification events took place between 0.35–0.53 Ma. In *Sancus acoreensis* the latter time point is placed further back to 1.12 Ma (Fig. 3). Therefore, it seems that there is a consensus in the time frame of diversification of all the arthropod species we investigated and this time frame is very close to or congruent with those reported from other animal or plant studies from the Azorean region. Nonetheless, there are also cases from which our findings deviate significantly. In their analyses of the plant genus *Festuca*, Diaz-Perez et al. [39] reported a time frame of diversification of 1.1 ± 0.6 Ma, whereas in *Pericallis* a diversification period of 3 Myr, (95 % confidence interval: 7.6–0.8 Ma) was estimated [27]. Rumeu et al. [40] estimated that *Juniperus brevifollia* has been diversifying within the Azores over the last 2.4–3.3 Myr (inferred from their Figs. 2 and 3). Ferreira et al. [35] estimated the divergence time of the Azorean endemic *Picconia azorica* and *P. excelsa* (endemic to the Madeira and Canary archipelagos), to be approximately 5 Ma, and thus claimed this to be the minimum age of the presence of *P. azorica* in the Azores. Finally, the time frame estimated for two groups of invertebrate species that include several species in the Azorean region, are quite different from each other and only one of them is congruent with our estimates. More specifically for the coleopteran species of *Tarphius* [26], the colonization of the Azores is reported to have occurred 7.42 Ma (6.38–8.12, 95 % HPD), whereas for the land-snail species of *Leptaxis* a differentiation time frame going back to only 1.81 ± 0.61 Ma is estimated [41]. In the case of *Leptaxis,* the authors presumed that the differentiation is older, but claim that the most probable reason for the relatively small time estimates of the diversification of the species in the island complex, is the fact that the environmental conditions have not favoured the colonization by land snails, as a result of destructive volcanic activity. Based on published geological data, they report that the most active, destructive periods of volcanism on the two oldest Azorean islands are dated to c. 4 Ma. The last eruptive episode in the genesis of Santa Maria occurred about 2 Ma, but massive volcanic activity in the two oldest, easternmost volcanic regions of São Miguel lasted until 0.65 Ma. Although Flores emerged around 2 Ma, the island may have only become available for colonization around 0.65 Ma. Furthermore, the formation of Terceira, the oldest central Azorean island (3.52 Ma), was driven by a complex series of explosive, volcanic eruptions, and destructive earthquakes that lasted until about 0.3 Ma, whereas the western islands, Corvo and Flores, were apparently colonized by *Leptaxis* by 0.89 ± 0.33 Ma ([41] and geological references therein). Given the foregoing, as highlighted in [20] the “youthfulness” (62 % of the total area of the Azores is less than 1 Myr old) of the Azorean archipelago is apparent, and serves as a possible explanation of the observed pattern of endemism in the Azores [20, 31]. Our estimation is simplistic since in many islands, e.g. Terceira, although parts of the island are younger than 1 Ma, for the whole area of the island we used 3.52 Ma. So, if a more accurate calculation of the land areas of all the islands was carried out, then the percentage would certainly surpass 70 %. The time estimates inferred from the phylogenetic analyses based on the mtDNA sequence analysed herein, also point to a very recent diversification pattern for all the studies analysed. The same conclusion is inferred from the population genetics and the network analyses of the studied species.

Our study supports the hypothesis that the relative youth of the Azorean islands, together with major volcanic destructive events occurring over time and until very recently, have not provided adequate time for the species to diversify on the islands, but rather only enough time for them to disperse within the archipelago. Having already occupied all the islands of the archipelago, several of the studied species started to expand and diversify *in situ* by taking advantage of the more favourable conditions prevailing in the islands following the termination of the intense volcanic activity. Depending on which Azorean islands one focuses on, the volcanic activity of the archipelago has ceased somewhere between 2.0 (Santa Maria) and 0.3 Ma (Terceira), or even more recently on Pico, and these time-frames provide a better fit to the time estimates of the major splitting events of our six species than the maximum island ages reported in the literature. For the majority of the studied species, most of these diversification events have taken place in the later part of the Pleistocene, roughly 0.35–0.53 Ma. However, considering the hypotheses described above, one cannot exclude the possibility of an ancestral stock of haplotypes having accumulated, diversifying for a time period much larger than inferred by our analyses, but having suffered episodes of extinction driven by volcanism.

If it is the case that extinctions have been frequent, then what we are actually reconstructing with the mtDNA sequence data, is the evolution of the species on the island complex during the last 2 Ma, using as a starting point some more recently derived haplotypes of the ancestral stock. The volcanic history of the Azores has been very intense and several ancestral taxa may have gone extinct in the past through natural processes [42]. More recent (last 600 years) population and probably species extinctions, through anthropogenic habitat destruction, have also occurred. In a recent study, Cardoso et al. [24] concluded that unrecorded extinctions of spider species in the oldest and more disturbed islands could explain current species richness patterns. Therefore, the ancestral haplotypes could have gone extinct either recently or in the past. However, our population genetics analyses, at least for *Sancus acoreensis* and *P. oromii,* are not in favour of recent extinctions, rather, recent expansion of populations is evident (Table 2). Then the question concerning all the species analysed is why has extinction targeted only the ancestral haplotype stock and not the derived haplotypes?

Most probably the extinctions involved all the haplotypes of a single island and not just the ancestral stock, and the colonization cycle was each time initiated from the nearest island that maintained derived haplotypes (often constituting back-colonization), thus moving the diversification age of the species in the island complex closer to the present. In this scenario, the most likely source of recolonization is Santa Maria, which was the sole land mass of the archipelago for at least 4 Myr, while from around 4 Ma to almost 0.5 Ma São Miguel was much smaller than it currently is [43]. Thus, as expressed in the intra-archipelagic missing stepping-stone hypothesis of [20] it is very likely that many of the lineages we are investigating diversified on Santa Maria during the 4 Myr when this island was the only land mass in the area. Other newly formed Azorean islands were then colonized by lineages that originally diversified in Santa Maria but for which derived haplotypes survived in those islands and not in Santa Maria. If we were to accept this scenario, we have also to recognize that there is evidence for a different scenario for some taxa, which have been able to maintain ancestral haplotype stock. An example is provided by the flightless *Tarphius* [26], which have diversified and speciated within the oldest island of the Azores, Santa Maria, producing at least four sympatric species. Another similar case of ancestral haplotype maintenance in one of the older islands is recorded for the land snail species *Oxychilus atlanticus* from São Miguel [44]. This species was estimated to have been differentiating in the island of São Miguel for the last 3.08–7.17 Myr, a time frame very close to the maximum geological age of the island [44]. The ability to maintain ancestral lineages in an archipelago with such intense volcanic activity seems to be taxon specific. The very low dispersal abilities of *Tarphius* beetles and most certainly of the *Oxychilus* land snail may explain the haplotype persistence they exhibited in the Azores. Land snails are notorious for being able to maintain ancestral polymorphisms precisely because of their highly structured populations [45]. With the exception of the beetle *A. dolosus*, species in our study have much higher dispersal abilities compared both to *Tarphius* beetles and *Oxychilus* land snails and we may speculate that they were more efficient in expanding their range within the Azores. The lack of population isolation, with gene flow occurring among the islands, would prevent differentiation and speciation in the long term, which is supported by the Φst and IBD findings for some of the studied species. However, with a small sample of just six species, we cannot evaluate the contribution of dispersal ability in the overtime persistence of haplotypes.

In archipelagos of volcanic origin, with a clear sequence of island emergence and ageing over time, a pattern that is commonly recorded involves the colonization of the newly emerging islands from populations originating from older islands [4]. This creates a progression rule pattern [1, 46]. In the few comprehensive phylogeographic studies that generated data from which such a pattern can be inferred or where it was explicitly investigated, it seems that the Azorean archipelago conforms to this rule. In a study of *Leptaxis* [41] the authors claimed that the radiation in the Azores started on the oldest island, Santa Maria and proceeded via the second oldest island, São Miguel and from there it followed a westward direction, to the much younger islands of the central island group, most likely via Terceira. A similar colonization route that moves progressively from the oldest island of Santa Maria to the younger ones, is recorded in the study of *Tarphius* [26]. For *Juniperus brevifollia* [40] the authors state that the key factor explaining the distribution of the plastid DNA variation is the sequence of island emergence. Finally Díaz-Pérez and colleagues [39] report two different colonization routes for different species of the same genus. More specifically, they support that the initial colonization of *Festuca francoi* could have occurred in the eastern part of the Azorean archipelago and followed a westward dispersal from São Miguel to the central islands and then from Terceira to Flores. However, for *Festuca petraea* two scenarios are put forward by the authors. The first one involves a bidirectional centripetal colonization from Faial or Graciosa going to São Jorge and Pico islands and from the central islands of Faial and Graciosa to Flores in the west. The second scenario, the most favoured by their data, involves Flores as the starting point of colonization.

In the six species studied herein, the CTMC model implementation in BEAST has generated results that in most cases are marginally supporting one island over the other as being the starting point of the colonization of the Azorean islands. In addition, the BSSVS analysis has been able to support several colonization events. Therefore, within the six species there is no general consensus regarding either the progression rule, or the intuitive east to west colonization direction. For most of the six species the islands of the central group participate in all the strongly supported colonization events inferred, supporting a recent evolutionary history for each species. This is particularly true for Faial, São Jorge, Terceira, and to a lesser degree for Pico, the most recent island. Second, it becomes apparent that between Flores, Santa Maria and São Miguel, the islands more involved in the diffusion of the genetic diversity within the island complex are Flores and to a lesser degree São Miguel. Santa Maria is less involved in this diffusion process, possibly due to extinction of ancestral stocks in this island. Finally, in the majority of the studied species it can be seen that colonization both from older to younger islands and from younger to older ones (back-colonization) has occurred. Hence, the progression rule gains some support, but the non-intuitive pattern of colonization involving a west to east direction, that was described for *Festuca petraea* [39], is also encountered.

**Fig. 8:**
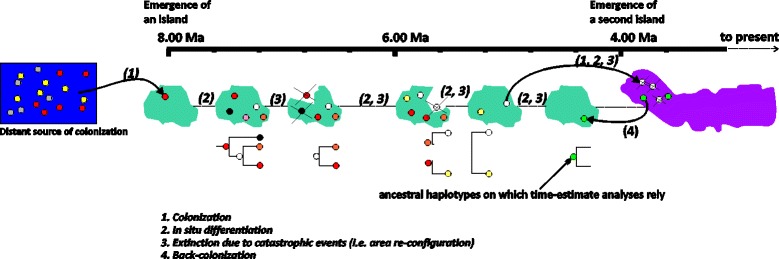
A schematic representation of the evolutionary history of the Azores as inferred from the studied arthropod species. For reasons of simplicity only two islands are represented. However, the model can be expanded to include all the islands of the group. The model proposes that following extinction due to catastrophic events, back-colonization processes occur and that together these perhaps reiterative processes shape the distribution of genetic diversity in the Azores and shorten the time-estimates inferred for the differentiation of the species within the archipelago

Given the geological history of the Azores, with many periods of intensive volcanic activity (sometimes involving catastrophic losses of terrain), it is reasonable to assume that extinction has played an important role in shaping the current phylogeographic patterns observed for the study species. Extinction may have been particularly important for the oldest islands, which were then re-colonized by individuals from nearby islands within the archipelago. In this case, the colonization events we inferred from younger to older island can be seen as back colonization events: an older island was re-colonized by lineages that survived and diversified on younger nearby islands, but originated from the ancestral lineages originally found on the older island (for a schematic representation of the proposed process see Fig. 8). Repeated cycles of extinction/re-colonization would push the diversification age of the species in the archipelago closer to the present, which is congruent with our results. Similarly, episodes of recent enlargement of area of an older island by new constructive volcanic episodes (as is a feature of Santa Maria for example), may also present opportunities for colonization of young terrain from other islands in the archipelago. The repeated re-colonization cycles would also explain why haplotype-rich islands (Table 2) are not inferred as the ancestral range of the species within the Azores. This is probably because their haplotype richness is the result of multiple colonization events and is not due to within island diversification.

At this point we have to acknowledge that our study relies on three linked mtDNA markers that are essentially non-independent markers. Therefore, we consider that the resolution of the obtained patterns could be significantly enhanced if nuclear markers were considered as well. However, the recent phylogeographic study of the species-rich Azorean beetle genus *Tarphius*, has shown that one of the most commonly used nuclear markers in arthropods, the elongation factor 1a (*Ef1a*) has provided a phylogeny that is significantly less resolved than its mtDNA counterpart [26]. Most likely nuclear markers with similar evolutionary rates to *Ef1a*, will not suffice. Consequently, in our view, future Azorean molecular studies dedicated to arthropods and perhaps in general to highly dispersive taxa, should aim to involve both mtDNA markers and very fast evolving nuclear markers such as microsatellites. This combination will allow the study of the distribution of the genetic diversity of the Azorean taxa over a time span that will be more representative of the actual *in-situ* differentiation time of the taxa in the Azores.

## Conclusions

Based on the levels of mtDNA diversity recorded in all the species, the prediction (3) that sequence diversity would be higher in the less dispersive species, is not verified. Very low levels of mtDNA sequence divergence are recorded both within and between islands, in all species. Furthermore, it seems that the sequence divergence recorded in each species, is not proportional to the geological ages of the Azorean Islands (prediction 1). Additionally, our findings do not support the prediction (2) that the oldest islands are the ones harbouring more genetic diversity compared to the recent ones. Interestingly, the islands with the highest number of haplotypes are not the same for each of the studied species. Only for two species, *G. occidentalis* and *Sancus acoreensis*, does it appear that Santa Maria, the oldest island of the archipelago, is the most haplotype-rich island, followed by the islands of the central group. In the remaining cases, the central group dominates in haplotype numbers. Regarding prediction 4, we found that all the species appear to have been differentiating in the Azores for a period ranging from 0.77 to 1.98 Myr, a time frame that significantly post-dates the origin of the Azores. A possible explanation for this is that the general young age of Azorean landmasses (62 % of the total area is less than 1 Ma) has not provided enough time for the species to diversify extensively within the islands, instead generating patterns featuring strong signals of inter-island dispersal. On the other hand, the extinction of the ancestral haplotype stock in some species could also account for the inconsistency observed between archipelago age and diversification time estimates. In the case that extinction is indeed a major driving force of the distribution of lineages in the archipelago, then it makes sense to assume that after each extinction event, the colonization cycle would be initiated from a nearby island (back-colonization) that maintained derived haplotypes, thus moving the diversification age of the species in the Azores closer to the present (Fig. 8). Furthermore, the islands of the central group participate in all the strongly supported migration routes inferred, whereas between Flores, Santa Maria and São Miguel, the islands more involved in the diffusion of the genetic diversity within the island complex, are Flores and to a lesser degree São Miguel. Santa Maria is less involved in the diffusion process. Finally, colonization events both from older to younger islands and vice-versa (back-colonization) are recorded. Therefore, our results are partly congruent with prediction 4, since the progression rule is partly supported, and the non-intuitive pattern of colonization involving a west to east direction, is also encountered. In conclusion, this paper raises a model invoking an important role for island ontogeny and for archipelago ontogeny as setting the stage for the evolutionary dynamics played out within the archipelago. By the emphasis of this model on extinction processes our findings provide a development within the framework provided by the general dynamic model of oceanic island biogeography [4]. Further analyses of more lineages will be necessary to provide critical scrutiny of the model developed herein.

## Methods

### Sample collection

In 2010, sixteen native forest fragments distributed across seven of the nine Azorean islands were sampled (Corvo and Graciosa were not sampled) following a standardized protocol for epigean and canopy arthropods (BALA protocol [29, 47, 48]. Altogether, these fragments represent most of the remaining native forest cover of the Azores [29, 47, 48]. Details concerning the fragments, sites/transects sampled and the sampling procedure (pitfall traps and canopy beating) are provided in Additional file 1: Table S1. All specimens were sorted and identified to the species level. The study species are the spiders *Gibbaranea occidentalis* Wunderlich, 1989 (Araneidae)*, Sancus acoreensis* (Wunderlich, 1992) (Tetragnathidae) and *Savigniorrhipis acoreensis* Wunderlich, 1992 (Linyphiidae), the hemipterans *Aphrodes hamiltoni* Quartau & Borges, 2003 (Cicadellidae) and *Pinalitus oromii* J. Ribes, 1992 (Miridae) and the coleopteran *Alestrus dolosus* (Crotch, 1867) (Elateridae). The majority of the specimens analysed in this study originated from the pitfall traps and the canopy beating collections. However, some specimens of *Sancus acoreensis* were collected by hand. All six species are forest specialists. Of the six genera studied, only the spider genus *Savigniorrhipis* is represented by two species in the Azores. When genetic data were being collected for this study, the rare and recently described *Savigniorrhipis topographicus,* reported only from the island of São Jorge [49], had not yet been discovered, therefore it is not included in our study. Additional information on the six species is provided in Additional file 1: Table S2.

### DNA extraction and sequence data generation

We extracted total genomic DNA from each specimen using three different protocols, depending on the species (details in Supporting Information File). The mitochondrial (mtDNA) genes selected to be amplified were the cytochrome oxidase subunit I (COI), the large and the small ribosomal subunit (16S rRNA and 12S rRNA, respectively). These genes were selected because: (1) they are a combination of fast and slowly evolving mtDNA genes, (2) they are among the most commonly used markers for population genetics and phylogeographic analyses of arthropod species, (3) several sequences of these genes are available in GenBank from other arthropod species, thus facilitating the design of group-specific primers, and (4) the levels of sequence divergence recorded in our species would be directly comparable to those reported for other arthropod taxa. Details on the primers and the PCR conditions used to amplify the targeted mtDNA genes are provided in Additional file 1: Table S3. Automated sequencing of both strands of each amplicon was performed in an automated sequencer (using Big- Dye terminator chemistry). The primers in the sequencing reactions were the same as in the PCR amplifications. The mtDNA sequences generated were viewed, edited and aligned (Clustal algorithm) using CodonCode Aligner v. 2.06 (Genecodes Corporation). The alignment was manually improved when deemed necessary. The authenticity of the mtDNA sequences and the homology to the targeted mtDNA genes were evaluated with a BLAST search in the NCBI genetic database (http://blast.ncbi.nlm.nih.gov/Blast.cgi). The possibility of having sequenced nuclear copies of the mtDNA genes, known as numts [50], was also evaluated through the careful scrutiny of the generated chromatographs, for signs of ‘double peaks’ and spurious stop codons. The double peaks are typical in the presence of numts contamination, thus their absence eliminated such a possibility [51]. Absence of stop codons is also in favour of not having sequenced numts.

### Sequence divergence and population genetic analyses

Estimates of average evolutionary divergence over all sequence pairs in each species and for each mtDNA fragment separately, were estimated using MEGA v.6 [52]. Since 16S rRNA and 12S rRNA exhibited very low variability, population genetics analyses were performed only with the COI sequence data. The number of haplotypes (*h*), the haplotype diversity (*Hd*) and the nucleotide diversity (*π*) were calculated for each species as a whole (i.e. archipelago scale) and per island using DnaSP 5.10 [53]. To correct for uneven sample sizes, the number of haplotype was standardized with a rarefaction procedure using the CONTRIB software (R. J. Petit, available at: https://www6.bordeaux-aquitaine.inra.fr/biogeco/Production-scientifique/Logiciels/Contrib-Permut/Contrib). This rarefied number of haplotypes (hereafter h_rar_) was calculated for each island for which we had at least five individuals of the target species. To detect possible departures from a constant population size, we calculated Tajima’s *D* [54] and Fu’s *F*_*S*_ [55] using ARLEQUIN 3.5 [56]. The number of simulated samples was set to 5000. Negative *D* and *F*_*S*_ values (statistically significant) suggest demographic expansion while positive values of *D* and *F*_*S*_ point to a recently bottlenecked population or diversifying selection.

We estimated pairwise values of genetic differentiation using the fixation index Φst computed in ARLEQUIN 3.5 [56]. The significance of Φst was assessed by 10,000 permutations and critical significance levels for multiple testing were corrected in agreement with SR Narum [57] using a sequential Benjamini-Yekutieli procedure [58]. To test for patterns of isolation-by-distance (IBD) within the Azores, distances between islands were plotted against genetic distance (using Φst /(1- Φst) following the recommendations of [59] and the significance of this relationship was tested with a Mantel test using a Monte Carlo permutation procedure with 999 permutations as implemented in the R package “vegan” [60]. Statistical parsimony networks were constructed using the computer program TCS version 1.21 [61] using default settings.

### Phylogeographic analyses and divergence times estimation

To infer the phylogeographic history of each studied species, we used the discrete-state continuous-time Markov chain (CTMC) model [38] of phylogeographic analysis as implemented in BEAST v.2 [62]. The CTMC phylogenetic-biogeographic model enables the simultaneous estimation of phylogenetic relationships, lineage divergence times, ancestral ranges, and migration rates between geographic locations using Bayesian MCMC inference [63]. Depending on the species analysed we used four to seven geographical states (locations) corresponding to the islands from which each species was sampled. Only specimens for which all three gene fragments were available were used in the analyses. In the phylogeographic analyses of each studied species the sequence data of the three mtDNA genes were concatenated and treated as a single evolving fragment. Different gene-wise partitions schemes were implemented in the preliminary analysis for each species. Although the topology of the single partition and the gene-wise partition analysis was almost identical in all species, the single partition analyses provided significantly lower likelihood values. Thus, we present only the results from the single partition analysis. In all BEAST analyses a constant population size was chosen, as recommended for single-species phylogenies. For the analysis of each species, the nucleotide substitution model implemented was the one suggested by Modeltest [64] based on the Akaike Information Criterion (AIC) [65]. No outgroups were used in the analyses. The best supported root position was inferred by BEAST. If no outgroup is included, BEAST will automatically sample rooted trees, and the most plausible root position for the data analyzed under a molecular clock model, is indicated.

In order to time-calibrate the tree the evolutionary rate of the mtDNA of arthropods was used (0.0115 substitutions/site/million years), following [32]. Regarding spider species, substitution rates two or even four times higher than the mean arthropod rate have been estimated [66, 67]. However, judging from the level of sequence divergence recorded in the spider species of the study, these higher rates are considered highly unlikely. The mean arthropod rate was set as a prior in the uncorrelated lognormal distribution implemented for the ucld.mean value of the BEAST analyses. In each analysis (one for each species) two independent runs were performed on different processors for a chain length (generations) of 50 × 10^6^ and parameters were sampled every 5000 generations. The two separate runs were then combined (following the removal of 10 % burn-in) using LogCombiner v.2 [62]. For each independent run, adequate sampling and convergence of the chains to stationarity or distribution were confirmed by inspection of the MCMC samples using Tracer v.1.6 [68]. The effective sample size (ESS) values of all parameters were well above 200, which is usually considered a sufficient level of sampling [69]. The sampled posterior trees were summarized using TreeAnnotator v.2 to generate a maximum clade credibility tree (maximum posterior probabilities) and calculate the median ages and 95 % highest posterior density (HPD) intervals for each node. For identifying those colonization events that could explain the diffusion process, Bayesian Stochastic Search Variable Selection (BSSVS, an extension of the discrete phylogeographic model) was used [38] and the asymmetric model was implemented. We used a value of 4 as a threshold for the Bayes Factors test in order to consider a rate as being adequately supported in the BSSVS analysis.

## Availability of supporting data

The sequence data generated and analyzed herein have been deposited in GenBank. See Additional file 1: Table S4.

## Additional file

Additional file 1:
**Further details on sample collection, DNA extraction and sequence data generation methods.** **Table S1.** Sampling details. **Table S2.** Worldwide distribution of the studied genera, dispersal ability and habitat type of the studied species. **Table S3.** Primer pairs used for the PCR amplification of the three mtDNA fragments. **Table S4.** Individual specimen codes, species of origin and GenBank accession numbers for the three mtDNA fragments. **Table S5.** Pairwise values of genetic differentiation of each studied species between the Azorean islands. **Table S6.** Sequence data variability for each species. **Figure S1.** Location and map of the Azores. The islands of Corvo and Graciosa were not sampled. **Figure S2.** Statistical parsimony networks. (DOCX 1067 kb)

## Abbreviations

BSSVS: Bayesian stochastic search variable selection
CTMC: continuous-time Markov chain
HPD: highest posterior density
Ma: million years ago
Myr: million years
