# Supplementary material for: Comparative phylogeography of endemic Azorean arthropods
Source: BMC Evol Biol. 2015 Nov 11;15:250. doi: 10.1186/s12862-015-0523-x (PMC4642780; doi:10.1186/s12862-015-0523-x)
Supplement: Additional file 1: — Further details on sample collection, DNA extraction and sequence data generation methods. Table S1. Sampling details. Table S2. Worldwide distribution of the studied genera, dispersal ability and habitat type of the studied species. Table S3. Primer pairs used for the PCR amplification of the three mtDNA fragments. Table S4. Individual specimen codes, species of origin and GenBank accession numbers for the three mtDNA fragments. Table S5. Pairwise values of genetic differentiation of each studied species between the Azorean islands. Table S6. Sequence data variability for each species. Figure S1. Location and map of the Azores. The islands of Corvo and Graciosa were not sampled. Figure S2. Statistical parsimony networks. (DOCX 1067 kb) [file 12862_2015_523_MOESM1_ESM.docx]

**Supporting Information File**

Parmakelis *et al*. Comparative phylogeography of endemic Azorean arthropods

**Methods**

*Sampling protocol*

Before collecting material from the Azorean forest remnants, sampling licences were obtained from the Environmental Services according to Portuguese legislation (Law No 9/2012/A of March 20/2012, with additional guides set in DRR No 20/2012/A of 5 November 2012). All animal handling was conducted following the Regional Legislative Decree 15/2012/A, of April 2nd, that establishes the legal framework of nature conservation and biodiversity.

For each sampled site, a transect of 150 m long and 5 m wide was established during the summer months (June to September) [1, 2]. Along each transect, soil arthropods were surveyed with a set of 15 modified pitfall traps, while arthropods from woody plant species were sampled using a beating tray. Modified pitfall traps consisted of plastic cups of 11.5 cm diameter and 5.5 cm depth, containing a non-attractive preservative solution, propylene glycol (a DNA preservative). Inside the main pitfall cup a smaller cup (3.5 cm diameter, 6 cm deep), fixed with glue in the centre, was filled with attractive TURQUIN solution, prepared mainly with dark beer and some preservatives [3]. The inner cup was closed with a lid in which there were small holes that permitted the smell of TURQUIN to diffuse. The traps were submerged into the soil (with the rim at the surface level) every 10 m and a plastic plate, placed about 5 cm above surface level and fixed to the ground by two pieces of wire, was used to divert rain water from entering into the trap. The traps remained in the field for two weeks. Canopy sampling was conducted during the period that pitfall traps remained in the field, when the vegetation was dry. A square of 5 m width was established every 15 m (10 squares in total per transect). In each square, a replicate of the three most abundant woody plant species was sampled. Therefore, in each transect, ten individuals of each of the three most abundant woody plant species were sampled. However, in some transects fewer than three woody plants were present and only those were considered. For each selected plant, a branch was chosen at random, a beating tray was placed underneath and five beatings were made using a stick. The beating tray consisted of a cloth-inverted pyramid of 1 m width and 60 cm depth attached to a plastic collection bag at the end [2].

*DNA extraction and sequence data generation*

Total genomic DNA was extracted from the specimens. DNA extraction was performed using different protocols, depending on the species. Two of the protocols included the use of two commercial DNA isolation kits, namely the Macherey-Nagel (Germany) kit and the Pure link Genomic DNA kit of Invitrogen (USA) following the manufacturer’s instructions. The third protocol was the CTAB 2x protocol as modified by [4]. The protocol selected for each species was determined following a preliminary comparison of the three protocols on a subset of specimens. The criterion for applying the specific DNA extraction protocol was the efficiency in DNA extraction judged by gel electrophoresis evaluation.

For the majority of the species, the universal mtDNA primers tested did not provide amplicons of good quality, therefore specific primers were designed for the targeted mtDNA markers. The sequences of the newly designed primers are provided in Table S3. In most cases, the newly designed primers amplified larger fragments compared to the expected size using the universal primers. In each species ultimately included in the study, several pairs of primers and PCR conditions were tested in order to amplify the three targeted mtDNA genes. For each species and mtDNA gene targeted a specific combination of primers was used (Table S3). Each PCR was performed in a volume of either 50 or 25 μL, where 1–2 μL of template DNA was mixed with 0.2 mM dNTPs, 0.4 mM of each primer, and five units of Taq Polymerase (Hytest, Finland) per μL. The concentration of the MgCl_2_ varied between 2.5 and 3.5 mM depending on the species and the marker amplified. Thermocycling was performed in either a MyCycler (Biorad) or a TProfessional (Biometra) thermocycler. The cycle programs typically comprised an initial denaturation step at 95 °C for 3 min, followed by 40 cycles of 15 s at 95 °C, 1.5 min at 40– 52 °C (depending on the primer combination), and 1 min at 72 °C. The cycling was ended with 10 min sequence extension at 72 °C. The annealing temperatures of the PCRs varied both between and within the different markers amplified and between the different species.

**Table S1.** Sampling details.

Azorean native forest fragments (and transects within fragments) sampled. The number of individuals sequenced per mtDNA fragment is provided per species for the three arthropod groups (Araneae, Hemiptera, Coleoptera). Numbers in parentheses correspond to the length (bp) of each mtDNA gene in the aligned dataset of each of the studied species.

| **Spider species** | | *Gibbaranea occidentalis* | | |  | *Sancus acoreensis* | | |  | *Savigniorrhipis acoreensis* | | |  |
| --- | --- | --- | --- | --- | --- | --- | --- | --- | --- | --- | --- | --- | --- |
| Island (abbreviation used in the analyses) | Laurisilva fragment (transects) | COI (530 bp) | 16S rRNA (525 bp) | 12S rRNA (521 bp) |  | COI (558 bp) | 16S rRNA (774 bp) | 12S rRNA (614 bp) |  | COI (527 bp) | 16S rRNA (656 bp) | 12S rRNA (429 bp) |  |
| *Faial (FAI)* | Caldeira do Faial (T10, T11) | 8 | 2 | 0 |  | 0 | 0 | 0 |  | 0 | 0 | 0 |  |
|  | Cabeço do Fogo l (T01, T03) | 8 | 6 | 6 |  | 6 | 2 | 3 |  | 7 | 7 | 7 |  |
| *Flores (FLO)* | Caldeiras Funda e Rasa (T06, T07) | 8 | 8 | 8 |  | 15 | 15 | 16 |  | 7 | 7 | 7 |  |
|  | Morro Alto e Pico da Sé (T08, T16) | 8 | 8 | 7 |  | 13 | 16 | 12 |  | 5 | 5 | 5 |  |
| *Pico (PIC)* | Caveiro (T08, T09) | 8 | 8 | 5 |  | 16 | 13 | 10 |  | 3 | 3 | 3 |  |
|  | Mistério da Prainha (T01, T10) | 8 | 4 | 7 |  | 16 | 14 | 10 |  | 4 | 4 | 4 |  |
| *Santa Maria (SMA)* | Pico Alto (T01, T03) | 8 | 8 | 8 |  | 14 | 7 | 4 |  | 5 | 5 | 5 |  |
| *São Jorge (SJG)* | Pico Pinheiro (T02, T09) | 8 | 2 | 3 |  | 9 | 8 | 2 |  | 4 | 4 | 4 |  |
|  | Topo (T06, T12) | 1 | 0 | 1 |  | 5 | 7 | 2 |  | 6 | 6 | 6 |  |
| *São Miguel (SMG)* | Graminhais (T03, T07) | 1 | 1 | 1 |  | 15 | 15 | 9 |  | 4 | 4 | 4 |  |
|  | Pico da Vara (T01, T04) | 8 | 6 | 7 |  | 14 | 14 | 7 |  | 3 | 3 | 3 |  |
| *Terceira (TER)* | Biscoito da Ferraria (T01, T02) | 8 | 7 | 8 |  | 11 | 12 | 9 |  | 3 | 3 | 3 |  |
|  | Pico Galhardo (T22, T33) | 8 | 8 | 7 |  | 12 | 10 | 5 |  | 8 | 8 | 8 |  |
|  | Serra de Santa Bárbara (T06, T11, T43, T46) | 8 | 7 | 4 |  | 26 | 18 | 7 |  | 11 | 11 | 11 |  |
|  | Terra Brava (T15, T18) | 8 | 8 | 4 |  | 8 | 7 | 6 |  | 6 | 6 | 6 |  |
| Total |  | 106 | 83 | 76 |  | 180 | 158 | 102 |  | 76 | 76 | 76 |  |
| **Hemipteran species** | | *Aphrodes hamiltoni* | | |  | *Pinalitus oromii* | | |  |  |  |  |  |
| Island (abbreviation used in the analyses) | Laurisilva fragment (transects ) | COI (732 bp) | 16S rRNA (445 bp) | 12S rRNA (503 bp) |  | COI (536 bp) | 16S rRNA (550 bp) | 12S rRNA (570 bp) |  |  |  |  |  |
| *Faial (FAI)* | Caldeira do Faial (T10, T11) | 6 | 7 | 4 |  | 4 | 0 | 1 |  |  |  |  |  |
|  | Cabeço do Fogo (T01, T03) | 5 | 5 | 3 |  | 8 | 5 | 4 |  |  |  |  |  |
| *Flores (FLO)* | Caldeiras Funda e Rasa (T06, T07) | 5 | 3 | 4 |  | 9 | 5 | 7 |  |  |  |  |  |
|  | Morro Alto e Pico da Sé (T08, T16) | 2 | 2 | 2 |  | 7 | 6 | 5 |  |  |  |  |  |
| *Pico (PIC)* | Caveiro (T08, T09) | 2 | 4 | 3 |  | 7 | 5 | 4 |  |  |  |  |  |
|  | Mistério da Prainha (T01, T10) | 12 | 4 | 6 |  | 6 | 6 | 4 |  |  |  |  |  |
| *Santa Maria (SMA)* | Pico Alto (T01, T03) | 4 | 3 | 3 |  | 7 | 6 | 6 |  |  |  |  |  |
| *São Jorge (SJG)* | Pico Pinheiro (T02, T09) | 7 | 2 | 0 |  | 5 | 0 | 5 |  |  |  |  |  |
|  | Topo (T06, T12) | 1 | 1 | 2 |  | 6 | 2 | 4 |  |  |  |  |  |
| *São Miguel (SMG)* | Graminhais (T03, T07) | 0 | 0 | 0 |  | 1 | 0 | 1 |  |  |  |  |  |
|  | Pico da Vara (T01, T04) | 0 | 0 | 0 |  | 4 | 4 | 3 |  |  |  |  |  |
| *Terceira (TER)* | Biscoito da Ferraria (T01, T02) | 0 | 0 | 0 |  | 6 | 0 | 6 |  |  |  |  |  |
|  | Pico Galhardo (T22, T33) | 4 | 4 | 3 |  | 7 | 6 | 7 |  |  |  |  |  |
|  | Serra de Santa Bárbara (T06, T11, T43, T46) | 5 | 5 | 4 |  | 9 | 6 | 9 |  |  |  |  |  |
|  | Terra Brava (T15, T18) | 5 | 5 | 3 |  | 10 | 5 | 8 |  |  |  |  |  |
| Total |  | 58 | 45 | 37 |  | 96 | 56 | 74 |  |  |  |  |  |
| **Coleopteran species** | | ***Alestrus dolosus*** | | |  |  |  |  |  |  |  |  |  |
| Island (abbreviation used in the analyses) | Laurisilva fragment (transect) | COI (534 bp) | 16S rRNA (550 bp) | 12S rRNA (411 bp) |  |  |  |  |  |  |  |  |  |
| *Faial (FAI)* | Caldeira do Faial (T10, T11) | 0 | 0 | 0 |  |  |  |  |  |  |  |  |  |
|  | Cabeço do Fogo (T01, T03) | 0 | 0 | 0 |  |  |  |  |  |  |  |  |  |
| *Flores (FLO)* | Caldeiras Funda e Rasa (T06, T07) | 5 | 3 | 6 |  |  |  |  |  |  |  |  |  |
|  | Morro Alto e Pico da Sé (T08, T16) | 0 | 0 | 0 |  |  |  |  |  |  |  |  |  |
| *Pico (PIC)* | Caveiro (T08, T09) | 4 | 3 | 6 |  |  |  |  |  |  |  |  |  |
|  | Mistério da Prainha (T01, T10) | 0 | 0 | 0 |  |  |  |  |  |  |  |  |  |
| *Santa Maria (SMA)* | Pico Alto (T01, T03) | 0 | 0 | 0 |  |  |  |  |  |  |  |  |  |
| *São Jorge (SJG)* | Pico Pinheiro (T02, T09) | 0 | 0 | 0 |  |  |  |  |  |  |  |  |  |
|  | Topo (T06, T12) | 0 | 0 | 0 |  |  |  |  |  |  |  |  |  |
| *São Miguel (SMG)* | Graminhais (T03, T07) | 8 | 7 | 6 |  |  |  |  |  |  |  |  |  |
|  | Pico da Vara (T01, T04) | 7 | 6 | 7 |  |  |  |  |  |  |  |  |  |
| *Terceira (TER)* | Biscoito da Ferraria (T01, T02) | 0 | 0 | 0 |  |  |  |  |  |  |  |  |  |
|  | Pico Galhardo (T22, T33) | 3 | 2 | 3 |  |  |  |  |  |  |  |  |  |
|  | Serra de Santa Bárbara (T06, T11, T43, T46) | 0 | 0 | 0 |  |  |  |  |  |  |  |  |  |
|  | Terra Brava (T15, T18) | 3 | 2 | 2 |  |  |  |  |  |  |  |  |  |
| Total |  | 30 | 23 | 30 |  |  |  |  |  |  |  |  |  |

**Table S2.** Worldwide distribution of studied genera, dispersal ability and habitat type of the studied species.

| Species | Habitat | Dispersal abilitiy | # of congeneric spp. worldwide | Other congeneric spp. present in the Azores | Genus distribution |
| --- | --- | --- | --- | --- | --- |
| *G. occidentalis* | Canopy | High | 9 | ---- | Asia/Azores/Canary/Europe |
| *Sancus acoreensis* | Canopy | High | 2 | ---- | Africa/Azores |
| *Savigniorrhipis acoreensis* | Canopy | High | 1 | *S. topographicus* | Azores |
| *A. hamiltoni* | Soil | Low | 13 | ---- | Africa/Asia/Azores/Canada/Canary/Europe/Madeira |
| *P. oromii* | Canopy | High | 16 | ---- | Asia/Azores/Canary/Europe/Madeira/N.America |
| *A. dolosus* | Soil | Low | 1 | ---- | Azores |

**Table S3.** Primer pairs used for the PCR amplification of the three mtDNA fragments.

| **Species** |  | **primers combination** | | | | |
| --- | --- | --- | --- | --- | --- | --- |
|  |  | COI |  | 16S rRNA |  | 12S rRNA |
| *G. occidentalis* |  | nancy/mt6 |  | 16S_479_461R_Heterop/16S_87_106F_Heterop |  | 12S_CRF/12S_CRR |
|  |  |  |  | 16S_BR_N12887/16S_AR_J13398 |  | Spider_12S_34_54F/Spider_12S_582_561R |
| *Sancus acoreensis* |  | nancy/mt6 |  | SancAcor50F/SancAcor562R/SancAcor687R |  | 12S_CRF/12S_CRR |
|  |  |  |  | 16S_BR_N12887/16S_AR_J13398 |  | Spider_12S_34_54F/Spider_12S_582_561R |
|  |  |  |  | 16S_Lin_743_724R/16S_Lin_91_71_F |  |  |
| *Savigniorrhipis acoreensis* |  | nancy/mt6 |  | 16S_Lin_743_724R/16S_Lin_91_71_F |  | Spider_12S_34_54F/Spider_12S_582_561R |
|  |  |  |  |  |  | 12s_CRF/12s_CRR |
| *A. hamiltoni* |  | nancy/LCOI1490 |  | LR_J_13017/ LR_N_13398 |  | Spider_12S_59_76F/Spider_12S_582_561R |
|  |  |  |  | 16S_BR_N12887/16S_AR_J13398 |  | 12S_CRF/12s_CRR |
|  |  |  |  | 16S_479_461R_Heterop/16S_87_106F_Heterop |  | SR_J_14199/SR_N_14594 |
| *P. oromii* |  | nancy/mt6 |  | LR_J_13017/16S_AR_J13398 |  | Spider_12S_59_76F/Spider_12S_582_561R |
|  |  |  |  | 16S_479_461R_Heterop/16S_87_106F_Heterop |  |  |
|  |  |  |  | 16S_BR_N12887/16S_AR_J13398 |  |  |
| *A. dolosus* |  | nancy/mt6 |  | 16S_556_537_R/16S_9_30_F |  | 12s_CRF/12s_CRR |
|  |  |  |  | 16S_BR_N12887/16S_AR_J13398 |  |  |
|  |  |  |  |  |  |  |
| **mtDNA gene fragment** |  | **primer name** |  | **primer sequence** |  |  |
| COI |  |  |  |  |  |  |
|  |  | LCOI1490 |  | GGTCAACAAATCATAAAGATATTGG |  |  |
|  |  | mt6 |  | GGAGGATTTGGAAATTGATTAGTTCC |  |  |
|  |  | nancy |  | CCCGGTAAAATTAAAATATAAACTTC |  |  |
| 16S rRNA |  |  |  |  |  |  |
|  |  | 16S_479_461R_Heterop |  | TACAAAGGTAGCATAATCA |  |  |
|  |  | 16S_556_537_R |  | CGTCGCCTGTTTAACAAAAA |  |  |
|  |  | 16S_87_106F_Heterop |  | ATCCAACATCGAGGTCGCAA |  |  |
|  |  | 16S_9_30_F |  | TTGAACTCAGATCACGTAAAAT |  |  |
|  |  | 16S_AR_J13398 |  | CGCCTGTTTAACAAAAACAT |  |  |
|  |  | 16S_BR_N12887 |  | CCGGTTTGAACTCAGATCATGT |  |  |
|  |  | 16S_Lin_743_724R |  | ATGGCRTCWCTRAANGGTTG |  |  |
|  |  | 16S_Lin_91_71_F |  | GTGCTAAGGTAGCATAATCAT |  |  |
|  |  | LR_J_13017 |  | TTACGCTGTTATCCTAA |  |  |
|  |  | SancAcor50F |  | CTTWCGRATTTGGATGTAGC |  |  |
|  |  | SancAcor562R |  | AGACGAYAAGACCCTATYR |  |  |
|  |  | SancAcor687R |  | TAAGGTAGCATAATCATT |  |  |
| 12S rRNA |  |  |  |  |  |  |
|  |  | 12S_CRF |  | GAGAGTGACGGGCGATATGT |  |  |
|  |  | 12s_CRR |  | AAACCAGGATTAGATACCCTATTAT |  |  |
|  |  | Spider_12S_34_54F |  | TGTTACGACTTACCTYATHTT |  |  |
|  |  | Spider_12S_582_561R |  | GTGCCAGCRGYCGCGGTTATAC |  |  |
|  |  | Spider_12S_59_76F |  | HRAGGGTGACGGGCGATA |  |  |
|  |  | SR_J_14199 |  | TACTATGTTACGACTTAT |  |  |
|  |  | SR_N_14594 |  | AAACTAGGATTAGATACCC |  |  |

**Table S4.** Individual specimen codes, species of origin and GenBank accession numbers for the three mtDNA fragments.

The sequences generated were all coded according to species, island and transect names. For example, a specimen of *Alestrus dolosus* from Flores island and transect T06 is coded Ad_FLO_T06. For the specimens collected by hand no transect code is given.

| **Individual specimen code** | **Species** | **Origin** |  | **Accession number** | | |
| --- | --- | --- | --- | --- | --- | --- |
|  |  |  |  | **COI** | **16S rRNA** | **12S rRNA** |
| Go_FAI_T01_E1_1 | *G. occidentalis* | Azores, Faial isl. |  | KP981737 |  |  |
| Go_FAI_T01_E5_1 | *G. occidentalis* | Azores, Faial isl. |  | KP981738 |  |  |
| Go_FAI_T01_E6_1 | *G. occidentalis* | Azores, Faial isl. |  | KP981739 |  |  |
| Go_FAI_T01_M1_1 | *G. occidentalis* | Azores, Faial isl. |  | KP981740 |  |  |
| Go_FAI_T02_J1_1 | *G. occidentalis* | Azores, Faial isl. |  | KP981741 |  |  |
| Go_FAI_T03_E10_1 | *G. occidentalis* | Azores, Faial isl. |  | KP981742 |  |  |
| Go_FAI_T03_E3_1 | *G. occidentalis* | Azores, Faial isl. |  | KP981743 |  |  |
| Go_FAI_T03_E5_1 | *G. occidentalis* | Azores, Faial isl. |  | KP981744 |  |  |
| Go_FAI_T03_E7_1 | *G. occidentalis* | Azores, Faial isl. |  | KP981745 |  |  |
| Go_FAI_T06_I9_1 | *G. occidentalis* | Azores, Faial isl. |  | KP981746 |  |  |
| Go_FAI_T10_E10_1 | *G. occidentalis* | Azores, Faial isl. |  | KP981747 | KP982244 | KP982571 |
| Go_FAI_T10_I1_1 | *G. occidentalis* | Azores, Faial isl. |  | KP981748 |  |  |
| Go_FAI_T10_I10_1 | *G. occidentalis* | Azores, Faial isl. |  | KP981749 | KP982245 | KP982572 |
| Go_FAI_T10_I6_1 | *G. occidentalis* | Azores, Faial isl. |  | KP981750 | KP982246 | KP982573 |
| Go_FAI_T10_L2_1 | *G. occidentalis* | Azores, Faial isl. |  | KP981751 | KP982247 | KP982574 |
| Go_FAI_T11_E5_1 | *G. occidentalis* | Azores, Faial isl. |  | KP981752 |  |  |
| Go_FAI_T11_E7_1 | *G. occidentalis* | Azores, Faial isl. |  | KP981753 | KP982248 | KP982575 |
| Go_FAI_T11_E9_1 | *G. occidentalis* | Azores, Faial isl. |  | KP981754 | KP982249 | KP982576 |
| Go_FLO_T06_J5_1 | *G. occidentalis* | Azores, Flores isl. |  | KP981755 | KP982250 | KP982577 |
| Go_FLO_T06_J7_1 | *G. occidentalis* | Azores, Flores isl. |  | KP981756 | KP982251 | KP982578 |
| Go_FLO_T06_Py15_1 | *G. occidentalis* | Azores, Flores isl. |  | KP981757 | KP982252 | KP982579 |
| Go_FLO_T06_Py7_1 | *G. occidentalis* | Azores, Flores isl. |  | KP981758 | KP982253 | KP982580 |
| Go_FLO_T07_2011_1 | *G. occidentalis* | Azores, Flores isl. |  | KP981759 | KP982254 | KP982581 |
| Go_FLO_T07_2011_2 | *G. occidentalis* | Azores, Flores isl. |  | KP981760 | KP982255 | KP982582 |
| Go_FLO_T07_I2_1 | *G. occidentalis* | Azores, Flores isl. |  | KP981761 | KP982256 | KP982583 |
| Go_FLO_T07_J9_1 | *G. occidentalis* | Azores, Flores isl. |  | KP981762 | KP982257 | KP982584 |
| Go_FLO_T08_2011_1 | *G. occidentalis* | Azores, Flores isl. |  | KP981763 | KP982258 | KP982585 |
| Go_FLO_T08_J1_1 | *G. occidentalis* | Azores, Flores isl. |  | KP981764 |  |  |
| Go_FLO_T08_J3_1 | *G. occidentalis* | Azores, Flores isl. |  | KP981765 | KP982259 | KP982586 |
| Go_FLO_T08_V3_1 | *G. occidentalis* | Azores, Flores isl. |  | KP981766 | KP982260 | KP982587 |
| Go_FLO_T16_I1_1 | *G. occidentalis* | Azores, Flores isl. |  | KP981767 | KP982261 | KP982588 |
| Go_FLO_T16_J1_1 | *G. occidentalis* | Azores, Flores isl. |  | KP981768 | KP982262 | KP982589 |
| Go_FLO_T16_J3_1 | *G. occidentalis* | Azores, Flores isl. |  | KP981769 | KP982263 | KP982590 |
| Go_FLO_T16_L9_1 | *G. occidentalis* | Azores, Flores isl. |  | KP981770 | KP982264 | KP982591 |
| Go_PIC_MP_2011_1 | *G. occidentalis* | Azores, Pico isl. |  | KP981771 |  |  |
| Go_PIC_MP_2011_2 | *G. occidentalis* | Azores, Pico isl. |  | KP981772 |  |  |
| Go_PIC_MP_2011_3 | *G. occidentalis* | Azores, Pico isl. |  | KP981773 |  |  |
| Go_PIC_MP_2011_4 | *G. occidentalis* | Azores, Pico isl. |  | KP981774 |  |  |
| Go_PIC_T01_I1_1a | *G. occidentalis* | Azores, Pico isl. |  | KP981775 | KP982265 | KP982592 |
| Go_PIC_T01_I3_1 | *G. occidentalis* | Azores, Pico isl. |  | KP981776 |  |  |
| Go_PIC_T01_L3_1a | *G. occidentalis* | Azores, Pico isl. |  | KP981777 | KP982266 | KP982593 |
| Go_PIC_T01_L9_1 | *G. occidentalis* | Azores, Pico isl. |  | KP981778 | KP982267 | KP982594 |
| Go_PIC_T08_I1_1 | *G. occidentalis* | Azores, Pico isl. |  | KP981779 |  |  |
| Go_PIC_T08_I6_1 | *G. occidentalis* | Azores, Pico isl. |  | KP981780 | KP982268 | KP982595 |
| Go_PIC_T08_I7_1 | *G. occidentalis* | Azores, Pico isl. |  | KP981781 |  |  |
| Go_PIC_T08_I8_1 | *G. occidentalis* | Azores, Pico isl. |  | KP981782 | KP982269 | KP982596 |
| Go_PIC_T09_I10_1a | *G. occidentalis* | Azores, Pico isl. |  | KP981783 | KP982270 | KP982597 |
| Go_PIC_T09_I5_1a | *G. occidentalis* | Azores, Pico isl. |  | KP981784 |  |  |
| Go_PIC_T09_J10_1 | *G. occidentalis* | Azores, Pico isl. |  | KP981785 | KP982271 | KP982598 |
| Go_PIC_T09_J9_1a | *G. occidentalis* | Azores, Pico isl. |  | KP981786 | KP982272 | KP982599 |
| Go_SJG_T02_J3_1 | *G. occidentalis* | Azores, São Jorge isl. |  | KP981787 |  |  |
| Go_SJG_T02_V10_1 | *G. occidentalis* | Azores, São Jorge isl. |  | KP981788 |  |  |
| Go_SJG_T02_V2_1 | *G. occidentalis* | Azores, São Jorge isl. |  | KP981789 |  |  |
| Go_SJG_T09_J2_1 | *G. occidentalis* | Azores, São Jorge isl. |  | KP981790 |  |  |
| Go_SJG_T09_J3_1 | *G. occidentalis* | Azores, São Jorge isl. |  | KP981791 | KP982273 | KP982600 |
| Go_SJG_T09_V4_1 | *G. occidentalis* | Azores, São Jorge isl. |  | KP981792 |  |  |
| Go_SJG_T09_V6_1 | *G. occidentalis* | Azores, São Jorge isl. |  | KP981793 | KP982274 | KP982601 |
| Go_SMA_T01_L10_1a | *G. occidentalis* | Azores, Santa Maria isl. |  | KP981794 | KP982275 | KP982602 |
| Go_SMA_T01_L4_1a | *G. occidentalis* | Azores, Santa Maria isl. |  | KP981795 | KP982276 | KP982603 |
| Go_SMA_T01_L6_1 | *G. occidentalis* | Azores, Santa Maria isl. |  | KP981796 | KP982277 | KP982604 |
| Go_SMA_T01_P5_1a | *G. occidentalis* | Azores, Santa Maria isl. |  | KP981797 | KP982278 | KP982605 |
| Go_SMA_T03_P7_1 | *G. occidentalis* | Azores, Santa Maria isl. |  | KP981798 | KP982279 | KP982606 |
| Go_SMA_T03_P8_1 | *G. occidentalis* | Azores, Santa Maria isl. |  | KP981799 | KP982280 | KP982607 |
| Go_SMA_T03_Pit1_1 | *G. occidentalis* | Azores, Santa Maria isl. |  | KP981800 | KP982281 | KP982608 |
| Go_SMA_T03_Pit2_1 | *G. occidentalis* | Azores, Santa Maria isl. |  | KP981801 | KP982282 | KP982609 |
| Go_SMG_T01_I10_1 | *G. occidentalis* | Azores, São Miguel isl. |  | KP981802 | KP982283 | KP982610 |
| Go_SMG_T01_I10_2 | *G. occidentalis* | Azores, São Miguel isl. |  | KP981803 | KP982284 | KP982611 |
| Go_SMG_T01_J1_1 | *G. occidentalis* | Azores, São Miguel isl. |  | KP981804 | KP982285 | KP982612 |
| Go_SMG_T03_L9_1 | *G. occidentalis* | Azores, São Miguel isl. |  | KP981805 | KP982286 | KP982613 |
| Go_SMG_T04_E9_1 | *G. occidentalis* | Azores, São Miguel isl. |  | KP981806 |  |  |
| Go_SMG_T04_I3_1 | *G. occidentalis* | Azores, São Miguel isl. |  | KP981807 | KP982287 | KP982614 |
| Go_SMG_T04_I5_1 | *G. occidentalis* | Azores, São Miguel isl. |  | KP981808 |  |  |
| Go_SMG_T04_L6_1 | *G. occidentalis* | Azores, São Miguel isl. |  | KP981809 | KP982288 | KP982615 |
| Go_SMG_TRO_2011_1 | *G. occidentalis* | Azores, São Miguel isl. |  | KP981810 |  |  |
| Go_TER_T01_I10_1 | *G. occidentalis* | Azores, Terceira isl. |  | KP981811 | KP982289 | KP982616 |
| Go_TER_T01_I3_1 | *G. occidentalis* | Azores, Terceira isl. |  | KP981812 | KP982290 | KP982617 |
| Go_TER_T01_I6_1 | *G. occidentalis* | Azores, Terceira isl. |  | KP981813 |  |  |
| Go_TER_T01_J3_1 | *G. occidentalis* | Azores, Terceira isl. |  | KP981814 | KP982291 | KP982618 |
| Go_TER_T02_J5_1 | *G. occidentalis* | Azores, Terceira isl. |  | KP981815 | KP982292 | KP982619 |
| Go_TER_T02_J7_1 | *G. occidentalis* | Azores, Terceira isl. |  | KP981816 | KP982293 | KP982620 |
| Go_TER_T02_J9_1 | *G. occidentalis* | Azores, Terceira isl. |  | KP981817 | KP982294 | KP982621 |
| Go_TER_T02_V6_1 | *G. occidentalis* | Azores, Terceira isl. |  | KP981818 | KP982295 | KP982622 |
| Go_TER_T06_I6_1 | *G. occidentalis* | Azores, Terceira isl. |  | KP981819 | KP982296 | KP982623 |
| Go_TER_T06_I9_1 | *G. occidentalis* | Azores, Terceira isl. |  | KP981820 | KP982297 | KP982624 |
| Go_TER_T06_J9_1 | *G. occidentalis* | Azores, Terceira isl. |  | KP981821 | KP982298 | KP982625 |
| Go_TER_T06_M4_1 | *G. occidentalis* | Azores, Terceira isl. |  | KP981822 | KP982299 | KP982626 |
| Go_TER_T11_I6_1 | *G. occidentalis* | Azores, Terceira isl. |  | KP981823 |  |  |
| Go_TER_T11_I7_1 | *G. occidentalis* | Azores, Terceira isl. |  | KP981824 |  |  |
| Go_TER_T11_I9_1 | *G. occidentalis* | Azores, Terceira isl. |  | KP981825 |  |  |
| Go_TER_T11_J1_1 | *G. occidentalis* | Azores, Terceira isl. |  | KP981826 |  |  |
| Go_TER_T15_J8_1a | *G. occidentalis* | Azores, Terceira isl. |  | KP981827 | KP982300 | KP982627 |
| Go_TER_T15_L10_1 | *G. occidentalis* | Azores, Terceira isl. |  | KP981828 | KP982301 | KP982628 |
| Go_TER_T15_L8_1 | *G. occidentalis* | Azores, Terceira isl. |  | KP981829 | KP982302 | KP982629 |
| Go_TER_T15_V5_1 | *G. occidentalis* | Azores, Terceira isl. |  | KP981830 | KP982303 | KP982630 |
| Go_TER_T18_I7_1 | *G. occidentalis* | Azores, Terceira isl. |  | KP981831 |  |  |
| Go_TER_T18_J2_1 | *G. occidentalis* | Azores, Terceira isl. |  | KP981832 |  |  |
| Go_TER_T18_J6_1 | *G. occidentalis* | Azores, Terceira isl. |  | KP981833 |  |  |
| Go_TER_T18_L6_1 | *G. occidentalis* | Azores, Terceira isl. |  | KP981834 |  |  |
| Go_TER_T22_J5_1 | *G. occidentalis* | Azores, Terceira isl. |  | KP981835 |  |  |
| Go_TER_T22_L5_1 | *G. occidentalis* | Azores, Terceira isl. |  | KP981836 | KP982304 | KP982631 |
| Go_TER_T22_V4_1a | *G. occidentalis* | Azores, Terceira isl. |  | KP981837 | KP982305 | KP982632 |
| Go_TER_T22_V7_1 | *G. occidentalis* | Azores, Terceira isl. |  | KP981838 | KP982306 | KP982633 |
| Go_TER_T33_J1_1 | *G. occidentalis* | Azores, Terceira isl. |  | KP981839 | KP982307 | KP982634 |
| Go_TER_T33_J4_1 | *G. occidentalis* | Azores, Terceira isl. |  | KP981840 | KP982308 | KP982635 |
| Go_TER_T33_J9_1 | *G. occidentalis* | Azores, Terceira isl. |  | KP981841 | KP982309 | KP982636 |
| Go_TER_T33_L5_1 | *G. occidentalis* | Azores, Terceira isl. |  | KP981842 | KP982310 | KP982637 |
| Sn_FAI_T10_I6_1 | *Sancus acoreensis* | Azores, Faial isl. |  | KP981939 |  |  |
| Sn_FAI_T10_I7_1 | *Sancus acoreensis* | Azores, Faial isl. |  | KP981940 | KP982352 | KP982679 |
| Sn_FAI_T10_J10_1 | *Sancus acoreensis* | Azores, Faial isl. |  | KP981941 |  |  |
| Sn_FAI_T10_J2_1 | *Sancus acoreensis* | Azores, Faial isl. |  | KP981942 |  |  |
| Sn_FAI_T10_L7_1 | *Sancus acoreensis* | Azores, Faial isl. |  | KP981943 |  |  |
| Sn_FAI_T11_E1_1 | *Sancus acoreensis* | Azores, Faial isl. |  | KP981944 | KP982353 | KP982680 |
| Sn_FLO_CFR_2011_1 | *Sancus acoreensis* | Azores, Flores isl. |  | KP981945 | KP982354 | KP982681 |
| Sn_FLO_CFR_2011_2 | *Sancus acoreensis* | Azores, Flores isl. |  | KP981946 | KP982355 | KP982682 |
| Sn_FLO_CFR_2011_3 | *Sancus acoreensis* | Azores, Flores isl. |  | KP981947 | KP982356 | KP982683 |
| Sn_FLO_CFR_2011_4 | *Sancus acoreensis* | Azores, Flores isl. |  | KP981948 | KP982357 | KP982684 |
| Sn_FLO_CFR_2011_5 | *Sancus acoreensis* | Azores, Flores isl. |  | KP981949 | KP982358 | KP982685 |
| Sn_FLO_CFR_2011_6 | *Sancus acoreensis* | Azores, Flores isl. |  | KP981950 | KP982359 | KP982686 |
| Sn_FLO_CFR_2011_7 | *Sancus acoreensis* | Azores, Flores isl. |  | KP981951 | KP982360 | KP982687 |
| Sn_FLO_MA_2011_2 | *Sancus acoreensis* | Azores, Flores isl. |  | KP981952 | KP982361 | KP982688 |
| Sn_FLO_T06_J5_1 | *Sancus acoreensis* | Azores, Flores isl. |  | KP981953 | KP982362 | KP982689 |
| Sn_FLO_T07_2011_1 | *Sancus acoreensis* | Azores, Flores isl. |  | KP981954 | KP982363 | KP982690 |
| Sn_FLO_T07_2011_2 | *Sancus acoreensis* | Azores, Flores isl. |  | KP981955 | KP982364 | KP982691 |
| Sn_FLO_T07_2011_3 | *Sancus acoreensis* | Azores, Flores isl. |  | KP981956 | KP982365 | KP982692 |
| Sn_FLO_T07_J2_1 | *Sancus acoreensis* | Azores, Flores isl. |  | KP981957 | KP982366 | KP982693 |
| Sn_FLO_T07_J3_1 | *Sancus acoreensis* | Azores, Flores isl. |  | KP981958 | KP982367 | KP982694 |
| Sn_FLO_T07_J4_1 | *Sancus acoreensis* | Azores, Flores isl. |  | KP981959 | KP982368 | KP982695 |
| Sn_FLO_T07_V9_1 | *Sancus acoreensis* | Azores, Flores isl. |  | KP981960 | KP982369 | KP982696 |
| Sn_FLO_T08_2011_1 | *Sancus acoreensis* | Azores, Flores isl. |  | KP981961 | KP982370 | KP982697 |
| Sn_FLO_T08_2011_2 | *Sancus acoreensis* | Azores, Flores isl. |  | KP981962 | KP982371 | KP982698 |
| Sn_FLO_T08_2011_3 | *Sancus acoreensis* | Azores, Flores isl. |  | KP981963 | KP982372 | KP982699 |
| Sn_FLO_T08_2011_4 | *Sancus acoreensis* | Azores, Flores isl. |  | KP981964 | KP982373 | KP982700 |
| Sn_FLO_T08_2011_6 | *Sancus acoreensis* | Azores, Flores isl. |  | KP981965 |  |  |
| Sn_FLO_T08_2011_8 | *Sancus acoreensis* | Azores, Flores isl. |  | KP981966 |  |  |
| Sn_FLO_T16_I3_1 | *Sancus acoreensis* | Azores, Flores isl. |  | KP981967 | KP982374 | KP982701 |
| Sn_FLO_T16_I4_1 | *Sancus acoreensis* | Azores, Flores isl. |  | KP981968 | KP982375 | KP982702 |
| Sn_FLO_T16_I4_2 | *Sancus acoreensis* | Azores, Flores isl. |  | KP981969 | KP982376 | KP982703 |
| Sn_FLO_T16_J3_1 | *Sancus acoreensis* | Azores, Flores isl. |  | KP981970 | KP982377 | KP982704 |
| Sn_FLO_T16_J3_2 | *Sancus acoreensis* | Azores, Flores isl. |  | KP981971 | KP982378 | KP982705 |
| Sn_FLO_T16_L4_1 | *Sancus acoreensis* | Azores, Flores isl. |  | KP981972 | KP982379 | KP982706 |
| Sn_PIC_T01_I10_1 | *Sancus acoreensis* | Azores, Pico isl. |  | KP981973 |  |  |
| Sn_PIC_T01_I3_1 | *Sancus acoreensis* | Azores, Pico isl. |  | KP981974 | KP982380 | KP982707 |
| Sn_PIC_T01_J2_1 | *Sancus acoreensis* | Azores, Pico isl. |  | KP981975 |  |  |
| Sn_PIC_T01_J6_1 | *Sancus acoreensis* | Azores, Pico isl. |  | KP981976 |  |  |
| Sn_PIC_T01_J7_1 | *Sancus acoreensis* | Azores, Pico isl. |  | KP981977 | KP982381 | KP982708 |
| Sn_PIC_T01_J9_1 | *Sancus acoreensis* | Azores, Pico isl. |  | KP981978 |  |  |
| Sn_PIC_T01_L2_1 | *Sancus acoreensis* | Azores, Pico isl. |  | KP981979 |  |  |
| Sn_PIC_T01_L2_2 | *Sancus acoreensis* | Azores, Pico isl. |  | KP981980 |  |  |
| Sn_PIC_T01_L7_1 | *Sancus acoreensis* | Azores, Pico isl. |  | KP981981 | KP982382 | KP982709 |
| Sn_PIC_T08_I1_1 | *Sancus acoreensis* | Azores, Pico isl. |  | KP981982 | KP982383 | KP982710 |
| Sn_PIC_T08_I10_1 | *Sancus acoreensis* | Azores, Pico isl. |  | KP981983 |  |  |
| Sn_PIC_T08_I2_1 | *Sancus acoreensis* | Azores, Pico isl. |  | KP981984 |  |  |
| Sn_PIC_T08_I7_1 | *Sancus acoreensis* | Azores, Pico isl. |  | KP981985 |  |  |
| Sn_PIC_T08_J4_1 | *Sancus acoreensis* | Azores, Pico isl. |  | KP981986 | KP982384 | KP982711 |
| Sn_PIC_T08_J6_1 | *Sancus acoreensis* | Azores, Pico isl. |  | KP981987 |  |  |
| Sn_PIC_T08_J8_1 | *Sancus acoreensis* | Azores, Pico isl. |  | KP981988 | KP982385 | KP982712 |
| Sn_PIC_T08_J9_1 | *Sancus acoreensis* | Azores, Pico isl. |  | KP981989 | KP982386 | KP982713 |
| Sn_PIC_T08_L10_1 | *Sancus acoreensis* | Azores, Pico isl. |  | KP981990 | KP982387 | KP982714 |
| Sn_PIC_T08_L4_1 | *Sancus acoreensis* | Azores, Pico isl. |  | KP981991 |  |  |
| Sn_PIC_T09_I4_1 | *Sancus acoreensis* | Azores, Pico isl. |  | KP981992 |  |  |
| Sn_PIC_T09_I5_1 | *Sancus acoreensis* | Azores, Pico isl. |  | KP981993 |  |  |
| Sn_PIC_T09_J7_1 | *Sancus acoreensis* | Azores, Pico isl. |  | KP981994 | KP982388 | KP982715 |
| Sn_PIC_T09_J8_1b | *Sancus acoreensis* | Azores, Pico isl. |  | KP981995 |  |  |
| Sn_PIC_T09_V5_1 | *Sancus acoreensis* | Azores, Pico isl. |  | KP981996 | KP982389 | KP982716 |
| Sn_PIC_T09_V6_1 | *Sancus acoreensis* | Azores, Pico isl. |  | KP981997 | KP982390 | KP982717 |
| Sn_PIC_T10_2011_1 | *Sancus acoreensis* | Azores, Pico isl. |  | KP981998 | KP982391 | KP982718 |
| Sn_PIC_T10_2011_2 | *Sancus acoreensis* | Azores, Pico isl. |  | KP981999 | KP982392 | KP982719 |
| Sn_PIC_T10_2011_3 | *Sancus acoreensis* | Azores, Pico isl. |  | KP982000 | KP982393 | KP982720 |
| Sn_PIC_T10_2011_4 | *Sancus acoreensis* | Azores, Pico isl. |  | KP982001 | KP982394 | KP982721 |
| Sn_PIC_T10_2011_5 | *Sancus acoreensis* | Azores, Pico isl. |  | KP982002 | KP982395 | KP982722 |
| Sn_PIC_T10_E8_1 | *Sancus acoreensis* | Azores, Pico isl. |  | KP982003 | KP982396 | KP982723 |
| Sn_PIC_T10_I4_1 | *Sancus acoreensis* | Azores, Pico isl. |  | KP982004 | KP982397 | KP982724 |
| Sn_PIC_T10_J2_1 | *Sancus acoreensis* | Azores, Pico isl. |  | KP982005 | KP982398 | KP982725 |
| Sn_SJG_T02_J5_1 | *Sancus acoreensis* | Azores, São Jorge isl. |  | KP982006 |  |  |
| Sn_SJG_T02_J9_1 | *Sancus acoreensis* | Azores, São Jorge isl. |  | KP982007 |  |  |
| Sn_SJG_T02_M3_1 | *Sancus acoreensis* | Azores, São Jorge isl. |  | KP982008 | KP982399 | KP982726 |
| Sn_SJG_T02_M5_1 | *Sancus acoreensis* | Azores, São Jorge isl. |  | KP982009 |  |  |
| Sn_SJG_T02_M6_1 | *Sancus acoreensis* | Azores, São Jorge isl. |  | KP982010 |  |  |
| Sn_SJG_T06_J6_1 | *Sancus acoreensis* | Azores, São Jorge isl. |  | KP982011 |  |  |
| Sn_SJG_T06_V9_1 | *Sancus acoreensis* | Azores, São Jorge isl. |  | KP982012 |  |  |
| Sn_SJG_T09_J5_1 | *Sancus acoreensis* | Azores, São Jorge isl. |  | KP982013 |  |  |
| Sn_SJG_T09_J8_1 | *Sancus acoreensis* | Azores, São Jorge isl. |  | KP982014 | KP982400 | KP982727 |
| Sn_SJG_T09_J9_1 | *Sancus acoreensis* | Azores, São Jorge isl. |  | KP982015 |  |  |
| Sn_SJG_T09_V8_1 | *Sancus acoreensis* | Azores, São Jorge isl. |  | KP982016 |  |  |
| Sn_SJG_T12_I4_1 | *Sancus acoreensis* | Azores, São Jorge isl. |  | KP982017 | KP982401 | KP982728 |
| Sn_SJG_T12_I8_1 | *Sancus acoreensis* | Azores, São Jorge isl. |  | KP982018 |  |  |
| Sn_SJG_T12_V3_1 | *Sancus acoreensis* | Azores, São Jorge isl. |  | KP982019 | KP982402 | KP982729 |
| Sn_SMA_T01_E1_1 | *Sancus acoreensis* | Azores, Santa Maria isl. |  | KP982020 |  |  |
| Sn_SMA_T01_L10_1 | *Sancus acoreensis* | Azores, Santa Maria isl. |  | KP982021 | KP982403 | KP982730 |
| Sn_SMA_T01_L2_1 | *Sancus acoreensis* | Azores, Santa Maria isl. |  | KP982022 |  |  |
| Sn_SMA_T01_P4_1 | *Sancus acoreensis* | Azores, Santa Maria isl. |  | KP982023 |  |  |
| Sn_SMA_T01_P7_2 | *Sancus acoreensis* | Azores, Santa Maria isl. |  | KP982024 |  |  |
| Sn_SMA_T01_P9_1 | *Sancus acoreensis* | Azores, Santa Maria isl. |  | KP982025 | KP982404 | KP982731 |
| Sn_SMA_T03_P1_1 | *Sancus acoreensis* | Azores, Santa Maria isl. |  | KP982026 |  |  |
| Sn_SMA_T03_P2_1 | *Sancus acoreensis* | Azores, Santa Maria isl. |  | KP982027 |  |  |
| Sn_SMA_T03_P3_1 | *Sancus acoreensis* | Azores, Santa Maria isl. |  | KP982028 | KP982405 | KP982732 |
| Sn_SMA_T03_P6_1 | *Sancus acoreensis* | Azores, Santa Maria isl. |  | KP982029 |  |  |
| Sn_SMA_T03_P8_1 | *Sancus acoreensis* | Azores, Santa Maria isl. |  | KP982030 | KP982406 | KP982733 |
| Sn_SMA_T03_Pit1_1 | *Sancus acoreensis* | Azores, Santa Maria isl. |  | KP982031 |  |  |
| Sn_SMA_T03_Pit3_1 | *Sancus acoreensis* | Azores, Santa Maria isl. |  | KP982032 |  |  |
| Sn_SMA_T03_Pit5_1 | *Sancus acoreensis* | Azores, Santa Maria isl. |  | KP982033 |  |  |
| Sn_SMG_GRA_2011_1 | *Sancus acoreensis* | Azores, São Miguel isl. |  | KP982034 | KP982407 | KP982734 |
| Sn_SMG_GRA_2011_2 | *Sancus acoreensis* | Azores, São Miguel isl. |  | KP982035 | KP982408 | KP982735 |
| Sn_SMG_GRA_2011_3 | *Sancus acoreensis* | Azores, São Miguel isl. |  | KP982036 | KP982409 | KP982736 |
| Sn_SMG_GRA_2011_4 | *Sancus acoreensis* | Azores, São Miguel isl. |  | KP982037 | KP982410 | KP982737 |
| Sn_SMG_GRA_2011_5 | *Sancus acoreensis* | Azores, São Miguel isl. |  | KP982038 | KP982411 | KP982738 |
| Sn_SMG_GRA_2011_6 | *Sancus acoreensis* | Azores, São Miguel isl. |  | KP982039 | KP982412 | KP982739 |
| Sn_SMG_GRA_2011_7 | *Sancus acoreensis* | Azores, São Miguel isl. |  | KP982040 | KP982413 | KP982740 |
| Sn_SMG_GRA_2011_8 | *Sancus acoreensis* | Azores, São Miguel isl. |  | KP982041 | KP982414 | KP982741 |
| Sn_SMG_T01_I1_1 | *Sancus acoreensis* | Azores, São Miguel isl. |  | KP982042 |  |  |
| Sn_SMG_T01_I2_1 | *Sancus acoreensis* | Azores, São Miguel isl. |  | KP982043 | KP982415 | KP982742 |
| Sn_SMG_T01_I6_1 | *Sancus acoreensis* | Azores, São Miguel isl. |  | KP982044 |  |  |
| Sn_SMG_T01_J1_1 | *Sancus acoreensis* | Azores, São Miguel isl. |  | KP982045 |  |  |
| Sn_SMG_T01_J7_1 | *Sancus acoreensis* | Azores, São Miguel isl. |  | KP982046 | KP982416 | KP982743 |
| Sn_SMG_T01_J9_1 | *Sancus acoreensis* | Azores, São Miguel isl. |  | KP982047 | KP982417 | KP982744 |
| Sn_SMG_T03_L4_1 | *Sancus acoreensis* | Azores, São Miguel isl. |  | KP982048 | KP982418 | KP982745 |
| Sn_SMG_T03_L4_2 | *Sancus acoreensis* | Azores, São Miguel isl. |  | KP982049 |  |  |
| Sn_SMG_T03_L9_1 | *Sancus acoreensis* | Azores, São Miguel isl. |  | KP982050 |  |  |
| Sn_SMG_T04_E7_1 | *Sancus acoreensis* | Azores, São Miguel isl. |  | KP982051 |  |  |
| Sn_SMG_T04_E8_1 | *Sancus acoreensis* | Azores, São Miguel isl. |  | KP982052 | KP982419 | KP982746 |
| Sn_SMG_T04_I3_1 | *Sancus acoreensis* | Azores, São Miguel isl. |  | KP982053 | KP982420 | KP982747 |
| Sn_SMG_T04_I5_1 | *Sancus acoreensis* | Azores, São Miguel isl. |  | KP982054 | KP982421 | KP982748 |
| Sn_SMG_T04_I7_2 | *Sancus acoreensis* | Azores, São Miguel isl. |  | KP982055 |  |  |
| Sn_SMG_T04_L5_1 | *Sancus acoreensis* | Azores, São Miguel isl. |  | KP982056 |  |  |
| Sn_SMG_T04_L6_1 | *Sancus acoreensis* | Azores, São Miguel isl. |  | KP982057 |  |  |
| Sn_SMG_T07_I1_1 | *Sancus acoreensis* | Azores, São Miguel isl. |  | KP982058 |  |  |
| Sn_SMG_T07_I3_1 | *Sancus acoreensis* | Azores, São Miguel isl. |  | KP982059 |  |  |
| Sn_SMG_T07_I7_1 | *Sancus acoreensis* | Azores, São Miguel isl. |  | KP982060 |  |  |
| Sn_SMG_T07_L7_1 | *Sancus acoreensis* | Azores, São Miguel isl. |  | KP982061 |  |  |
| Sn_TER_T01_I5_1 | *Sancus acoreensis* | Azores, Terceira isl. |  | KP982062 |  |  |
| Sn_TER_T01_I8_1 | *Sancus acoreensis* | Azores, Terceira isl. |  | KP982063 | KP982422 | KP982749 |
| Sn_TER_T01_J2_1 | *Sancus acoreensis* | Azores, Terceira isl. |  | KP982064 | KP982423 | KP982750 |
| Sn_TER_T01_J8_1 | *Sancus acoreensis* | Azores, Terceira isl. |  | KP982065 | KP982424 | KP982751 |
| Sn_TER_T01_J9_1 | *Sancus acoreensis* | Azores, Terceira isl. |  | KP982066 | KP982425 | KP982752 |
| Sn_TER_T01_L8_1 | *Sancus acoreensis* | Azores, Terceira isl. |  | KP982067 |  |  |
| Sn_TER_T02_2011_1 | *Sancus acoreensis* | Azores, Terceira isl. |  | KP982068 | KP982426 | KP982753 |
| Sn_TER_T02_M10_1 | *Sancus acoreensis* | Azores, Terceira isl. |  | KP982069 | KP982427 | KP982754 |
| Sn_TER_T02_M3_1 | *Sancus acoreensis* | Azores, Terceira isl. |  | KP982070 | KP982428 | KP982755 |
| Sn_TER_T02_M6_1 | *Sancus acoreensis* | Azores, Terceira isl. |  | KP982071 | KP982429 | KP982756 |
| Sn_TER_T02_V1_1 | *Sancus acoreensis* | Azores, Terceira isl. |  | KP982072 | KP982430 | KP982757 |
| Sn_TER_T06_I10_1 | *Sancus acoreensis* | Azores, Terceira isl. |  | KP982073 |  |  |
| Sn_TER_T06_I5_1 | *Sancus acoreensis* | Azores, Terceira isl. |  | KP982074 |  |  |
| Sn_TER_T06_J3_1 | *Sancus acoreensis* | Azores, Terceira isl. |  | KP982075 |  |  |
| Sn_TER_T06_J7_1 | *Sancus acoreensis* | Azores, Terceira isl. |  | KP982076 |  |  |
| Sn_TER_T06_J9_1 | *Sancus acoreensis* | Azores, Terceira isl. |  | KP982077 |  |  |
| Sn_TER_T06_M10_1 | *Sancus acoreensis* | Azores, Terceira isl. |  | KP982078 |  |  |
| Sn_TER_T06_M7_1 | *Sancus acoreensis* | Azores, Terceira isl. |  | KP982079 |  |  |
| Sn_TER_T06_M8_1 | *Sancus acoreensis* | Azores, Terceira isl. |  | KP982080 |  |  |
| Sn_TER_T11_I8_1 | *Sancus acoreensis* | Azores, Terceira isl. |  | KP982081 |  |  |
| Sn_TER_T11_J1_1 | *Sancus acoreensis* | Azores, Terceira isl. |  | KP982082 | KP982431 | KP982758 |
| Sn_TER_T11_J3_1 | *Sancus acoreensis* | Azores, Terceira isl. |  | KP982083 | KP982432 | KP982759 |
| Sn_TER_T11_J6_1 | *Sancus acoreensis* | Azores, Terceira isl. |  | KP982084 |  |  |
| Sn_TER_T11_J8_1 | *Sancus acoreensis* | Azores, Terceira isl. |  | KP982085 |  |  |
| Sn_TER_T11_L1_1 | *Sancus acoreensis* | Azores, Terceira isl. |  | KP982086 |  |  |
| Sn_TER_T11_L5_1 | *Sancus acoreensis* | Azores, Terceira isl. |  | KP982087 |  |  |
| Sn_TER_T15_I1_1 | *Sancus acoreensis* | Azores, Terceira isl. |  | KP982088 |  |  |
| Sn_TER_T15_J1_1 | *Sancus acoreensis* | Azores, Terceira isl. |  | KP982089 | KP982433 | KP982760 |
| Sn_TER_T15_L3_1 | *Sancus acoreensis* | Azores, Terceira isl. |  | KP982090 | KP982434 | KP982761 |
| Sn_TER_T18_I1_1 | *Sancus acoreensis* | Azores, Terceira isl. |  | KP982091 |  |  |
| Sn_TER_T18_I3_1 | *Sancus acoreensis* | Azores, Terceira isl. |  | KP982092 |  |  |
| Sn_TER_T18_I4_1 | *Sancus acoreensis* | Azores, Terceira isl. |  | KP982093 |  |  |
| Sn_TER_T18_J10_1 | *Sancus acoreensis* | Azores, Terceira isl. |  | KP982094 | KP982435 | KP982762 |
| Sn_TER_T18_J4_1 | *Sancus acoreensis* | Azores, Terceira isl. |  | KP982095 |  |  |
| Sn_TER_T22_J2_1 | *Sancus acoreensis* | Azores, Terceira isl. |  | KP982096 |  |  |
| Sn_TER_T22_J4_1 | *Sancus acoreensis* | Azores, Terceira isl. |  | KP982097 |  |  |
| Sn_TER_T22_J6_1 | *Sancus acoreensis* | Azores, Terceira isl. |  | KP982098 |  |  |
| Sn_TER_T22_J8_1 | *Sancus acoreensis* | Azores, Terceira isl. |  | KP982099 | KP982436 | KP982763 |
| Sn_TER_T22_V10_1 | *Sancus acoreensis* | Azores, Terceira isl. |  | KP982100 | KP982437 | KP982764 |
| Sn_TER_T22_V7_1 | *Sancus acoreensis* | Azores, Terceira isl. |  | KP982101 |  |  |
| Sn_TER_T22_V8_1 | *Sancus acoreensis* | Azores, Terceira isl. |  | KP982102 |  |  |
| Sn_TER_T33_J10_1 | *Sancus acoreensis* | Azores, Terceira isl. |  | KP982103 | KP982438 | KP982765 |
| Sn_TER_T33_J7_1 | *Sancus acoreensis* | Azores, Terceira isl. |  | KP982104 | KP982439 | KP982766 |
| Sn_TER_T33_J9_1 | *Sancus acoreensis* | Azores, Terceira isl. |  | KP982105 |  |  |
| Sn_TER_T33_V7_1 | *Sancus acoreensis* | Azores, Terceira isl. |  | KP982106 | KP982440 | KP982767 |
| Sn_TER_T33_V8_1 | *Sancus acoreensis* | Azores, Terceira isl. |  | KP982107 |  |  |
| Sn_TER_T43_2011_1 | *Sancus acoreensis* | Azores, Terceira isl. |  | KP982108 |  |  |
| Sn_TER_T43_2011_2 | *Sancus acoreensis* | Azores, Terceira isl. |  | KP982109 |  |  |
| Sn_TER_T43_2011_3 | *Sancus acoreensis* | Azores, Terceira isl. |  | KP982110 |  |  |
| Sn_TER_T43_2011_4 | *Sancus acoreensis* | Azores, Terceira isl. |  | KP982111 |  |  |
| Sn_TER_T43_2011_5 | *Sancus acoreensis* | Azores, Terceira isl. |  | KP982112 | KP982441 | KP982768 |
| Sn_TER_T43_2011_6 | *Sancus acoreensis* | Azores, Terceira isl. |  | KP982113 | KP982442 | KP982769 |
| Sn_TER_T46_2011_1 | *Sancus acoreensis* | Azores, Terceira isl. |  | KP982114 | KP982443 | KP982770 |
| Sn_TER_T46_2011_2 | *Sancus acoreensis* | Azores, Terceira isl. |  | KP982115 |  |  |
| Sn_TER_T46_2011_3 | *Sancus acoreensis* | Azores, Terceira isl. |  | KP982116 |  |  |
| Sn_TER_T46_2011_4 | *Sancus acoreensis* | Azores, Terceira isl. |  | KP982117 | KP982444 | KP982771 |
| Sn_TER_T46_2011_5 | *Sancus acoreensis* | Azores, Terceira isl. |  | KP982118 | KP982445 | KP982772 |
| SaFAIT10J1_1 | *Savigniorrhipis acoreensis* | Azores, Faial isl. |  | KP982119 | KP982446 | KP982773 |
| SaFAIT10J1_2 | *Savigniorrhipis acoreensis* | Azores, Faial isl. |  | KP982120 | KP982447 | KP982774 |
| SaFAIT10L1_1 | *Savigniorrhipis acoreensis* | Azores, Faial isl. |  | KP982121 | KP982448 | KP982775 |
| SaFAIT10L3_1 | *Savigniorrhipis acoreensis* | Azores, Faial isl. |  | KP982122 | KP982449 | KP982776 |
| SaFAIT11E4_1 | *Savigniorrhipis acoreensis* | Azores, Faial isl. |  | KP982123 | KP982450 | KP982777 |
| SaFAIT11E8_1 | *Savigniorrhipis acoreensis* | Azores, Faial isl. |  | KP982124 | KP982451 | KP982778 |
| SaFAIT11E9_1 | *Savigniorrhipis acoreensis* | Azores, Faial isl. |  | KP982125 | KP982452 | KP982779 |
| SaFLOT06J5_1 | *Savigniorrhipis acoreensis* | Azores, Flores isl. |  | KP982126 | KP982453 | KP982780 |
| SaFLOT06J8_1 | *Savigniorrhipis acoreensis* | Azores, Flores isl. |  | KP982127 | KP982454 | KP982781 |
| SaFLOT06J9_1 | *Savigniorrhipis acoreensis* | Azores, Flores isl. |  | KP982128 | KP982455 | KP982782 |
| SaFLOT07I3_1 | *Savigniorrhipis acoreensis* | Azores, Flores isl. |  | KP982129 | KP982456 | KP982783 |
| SaFLOT07I7_1 | *Savigniorrhipis acoreensis* | Azores, Flores isl. |  | KP982130 | KP982457 | KP982784 |
| SaFLOT07J2_1 | *Savigniorrhipis acoreensis* | Azores, Flores isl. |  | KP982131 | KP982458 | KP982785 |
| SaFLOT07V10_1 | *Savigniorrhipis acoreensis* | Azores, Flores isl. |  | KP982132 | KP982459 | KP982786 |
| SaFLOT08V10_1 | *Savigniorrhipis acoreensis* | Azores, Flores isl. |  | KP982133 | KP982460 | KP982787 |
| SaFLOT08V9_1 | *Savigniorrhipis acoreensis* | Azores, Flores isl. |  | KP982134 | KP982461 | KP982788 |
| SaFLOT16J6_1 | *Savigniorrhipis acoreensis* | Azores, Flores isl. |  | KP982135 | KP982462 | KP982789 |
| SaFLOT16L1_1 | *Savigniorrhipis acoreensis* | Azores, Flores isl. |  | KP982136 | KP982463 | KP982790 |
| SaFLOT16P5_1 | *Savigniorrhipis acoreensis* | Azores, Flores isl. |  | KP982137 | KP982464 | KP982791 |
| SaPICT01J4_1 | *Savigniorrhipis acoreensis* | Azores, Pico isl. |  | KP982138 | KP982465 | KP982792 |
| SaPICT08I9_1 | *Savigniorrhipis acoreensis* | Azores, Pico isl. |  | KP982139 | KP982466 | KP982793 |
| SaPICT08L9_1 | *Savigniorrhipis acoreensis* | Azores, Pico isl. |  | KP982140 | KP982467 | KP982794 |
| SaPICT09I4_1 | *Savigniorrhipis acoreensis* | Azores, Pico isl. |  | KP982141 | KP982468 | KP982795 |
| SaPICT10E7_1 | *Savigniorrhipis acoreensis* | Azores, Pico isl. |  | KP982142 | KP982469 | KP982796 |
| SaPICT10I6_1 | *Savigniorrhipis acoreensis* | Azores, Pico isl. |  | KP982143 | KP982470 | KP982797 |
| SaPICT10J8_1 | *Savigniorrhipis acoreensis* | Azores, Pico isl. |  | KP982144 | KP982471 | KP982798 |
| SaSJGT02M7_1 | *Savigniorrhipis acoreensis* | Azores, São Jorge isl. |  | KP982145 | KP982472 | KP982799 |
| SaSJGT02M9_1 | *Savigniorrhipis acoreensis* | Azores, São Jorge isl. |  | KP982146 | KP982473 | KP982800 |
| SaSJGT06I4_1 | *Savigniorrhipis acoreensis* | Azores, São Jorge isl. |  | KP982147 | KP982474 | KP982801 |
| SaSJGT06I4_2 | *Savigniorrhipis acoreensis* | Azores, São Jorge isl. |  | KP982148 | KP982475 | KP982802 |
| SaSJGT06I8_1 | *Savigniorrhipis acoreensis* | Azores, São Jorge isl. |  | KP982149 | KP982476 | KP982803 |
| SaSJGT06J6_1 | *Savigniorrhipis acoreensis* | Azores, São Jorge isl. |  | KP982150 | KP982477 | KP982804 |
| SaSJGT09V5_1 | *Savigniorrhipis acoreensis* | Azores, São Jorge isl. |  | KP982151 | KP982478 | KP982805 |
| SaSJGT09V6_1 | *Savigniorrhipis acoreensis* | Azores, São Jorge isl. |  | KP982152 | KP982479 | KP982806 |
| SaSJGT12J2_1 | *Savigniorrhipis acoreensis* | Azores, São Jorge isl. |  | KP982153 | KP982480 | KP982807 |
| SaSJGT12J6_1 | *Savigniorrhipis acoreensis* | Azores, São Jorge isl. |  | KP982154 | KP982481 | KP982808 |
| SaSMAT01P1_1 | *Savigniorrhipis acoreensis* | Azores, Santa Maria isl. |  | KP982155 | KP982482 | KP982809 |
| SaSMAT01P8_1 | *Savigniorrhipis acoreensis* | Azores, Santa Maria isl. |  | KP982156 | KP982483 | KP982810 |
| SaSMAT03P3_1 | *Savigniorrhipis acoreensis* | Azores, Santa Maria isl. |  | KP982157 | KP982484 | KP982811 |
| SaSMAT03P9_1 | *Savigniorrhipis acoreensis* | Azores, Santa Maria isl. |  | KP982158 | KP982485 | KP982812 |
| SaSMAT03Pit8_1 | *Savigniorrhipis acoreensis* | Azores, Santa Maria isl. |  | KP982159 | KP982486 | KP982813 |
| SaSMGT01J6_1 | *Savigniorrhipis acoreensis* | Azores, São Miguel isl. |  | KP982160 | KP982487 | KP982814 |
| SaSMGT01L5_1 | *Savigniorrhipis acoreensis* | Azores, São Miguel isl. |  | KP982161 | KP982488 | KP982815 |
| SaSMGT03J9_1 | *Savigniorrhipis acoreensis* | Azores, São Miguel isl. |  | KP982162 | KP982489 | KP982816 |
| SaSMGT03V6_1 | *Savigniorrhipis acoreensis* | Azores, São Miguel isl. |  | KP982163 | KP982490 | KP982817 |
| SaSMGT04E7_1 | *Savigniorrhipis acoreensis* | Azores, São Miguel isl. |  | KP982164 | KP982491 | KP982818 |
| SaSMGT07I4_1 | *Savigniorrhipis acoreensis* | Azores, São Miguel isl. |  | KP982165 | KP982492 | KP982819 |
| SaSMGT07V8_1 | *Savigniorrhipis acoreensis* | Azores, São Miguel isl. |  | KP982166 | KP982493 | KP982820 |
| SaT11J10_1 | *Savigniorrhipis acoreensis* | Azores, Terceira isl. |  | KP982167 | KP982494 | KP982821 |
| SaT15L8_1 | *Savigniorrhipis acoreensis* | Azores, Terceira isl. |  | KP982168 | KP982495 | KP982822 |
| SaT33J6_1 | *Savigniorrhipis acoreensis* | Azores, Terceira isl. |  | KP982169 | KP982496 | KP982823 |
| SaT33J6_2 | *Savigniorrhipis acoreensis* | Azores, Terceira isl. |  | KP982170 | KP982497 | KP982824 |
| SaTERT01I3_1 | *Savigniorrhipis acoreensis* | Azores, Terceira isl. |  | KP982171 | KP982498 | KP982825 |
| SaTERT02J1_1 | *Savigniorrhipis acoreensis* | Azores, Terceira isl. |  | KP982172 | KP982499 | KP982826 |
| SaTERT02J5_1 | *Savigniorrhipis acoreensis* | Azores, Terceira isl. |  | KP982173 | KP982500 | KP982827 |
| SaTERT06I3_1 | *Savigniorrhipis acoreensis* | Azores, Terceira isl. |  | KP982174 | KP982501 | KP982828 |
| SaTERT06I9_1 | *Savigniorrhipis acoreensis* | Azores, Terceira isl. |  | KP982175 | KP982502 | KP982829 |
| SaTERT06J2_1 | *Savigniorrhipis acoreensis* | Azores, Terceira isl. |  | KP982176 | KP982503 | KP982830 |
| SaTERT06J9_1 | *Savigniorrhipis acoreensis* | Azores, Terceira isl. |  | KP982177 | KP982504 | KP982831 |
| SaTERT11I3_1 | *Savigniorrhipis acoreensis* | Azores, Terceira isl. |  | KP982178 | KP982505 | KP982832 |
| SaTERT11J8_2 | *Savigniorrhipis acoreensis* | Azores, Terceira isl. |  | KP982179 | KP982506 | KP982833 |
| SaTERT11L5_1 | *Savigniorrhipis acoreensis* | Azores, Terceira isl. |  | KP982180 | KP982507 | KP982834 |
| SaTERT15J3_1 | *Savigniorrhipis acoreensis* | Azores, Terceira isl. |  | KP982181 | KP982508 | KP982835 |
| SaTERT15J8_1 | *Savigniorrhipis acoreensis* | Azores, Terceira isl. |  | KP982182 | KP982509 | KP982836 |
| SaTERT15L8_2 | *Savigniorrhipis acoreensis* | Azores, Terceira isl. |  | KP982183 | KP982510 | KP982837 |
| SaTERT18I10_1 | *Savigniorrhipis acoreensis* | Azores, Terceira isl. |  | KP982184 | KP982511 | KP982838 |
| SaTERT18L1_1 | *Savigniorrhipis acoreensis* | Azores, Terceira isl. |  | KP982185 | KP982512 | KP982839 |
| SaTERT22J1_1 | *Savigniorrhipis acoreensis* | Azores, Terceira isl. |  | KP982186 | KP982513 | KP982840 |
| SaTERT22J10_1 | *Savigniorrhipis acoreensis* | Azores, Terceira isl. |  | KP982187 | KP982514 | KP982841 |
| SaTERT22J10_2 | *Savigniorrhipis acoreensis* | Azores, Terceira isl. |  | KP982188 | KP982515 | KP982842 |
| SaTERT33J10_1 | *Savigniorrhipis acoreensis* | Azores, Terceira isl. |  | KP982189 | KP982516 | KP982843 |
| SaTERT33L1_1 | *Savigniorrhipis acoreensis* | Azores, Terceira isl. |  | KP982190 | KP982517 | KP982844 |
| SaTERT43_2 | *Savigniorrhipis acoreensis* | Azores, Terceira isl. |  | KP982191 | KP982518 | KP982845 |
| SaTERT43_3 | *Savigniorrhipis acoreensis* | Azores, Terceira isl. |  | KP982192 | KP982519 | KP982846 |
| SaTERT46_1 | *Savigniorrhipis acoreensis* | Azores, Terceira isl. |  | KP982193 | KP982520 | KP982847 |
| SaTERT46_2 | *Savigniorrhipis acoreensis* | Azores, Terceira isl. |  | KP982194 | KP982521 | KP982848 |
| AhFAIT01P11_1 | *A. hamiltoni* | Azores, Faial isl. |  | KP981679 | KP982214 | KP982541 |
| AhFAIT01P12_1 | *A. hamiltoni* | Azores, Faial isl. |  | KP981680 |  |  |
| AhFAIT03P1_1 | *A. hamiltoni* | Azores, Faial isl. |  | KP981681 | KP982215 | KP982542 |
| AhFAIT03P10_1 | *A. hamiltoni* | Azores, Faial isl. |  | KP981682 | KP982216 | KP982543 |
| AhFAIT03P4_1 | *A. hamiltoni* | Azores, Faial isl. |  | KP981683 | KP982217 | KP982544 |
| AhFAIT03P6_2 | *A. hamiltoni* | Azores, Faial isl. |  | KP981684 |  |  |
| AhFAIT10P12_1 | *A. hamiltoni* | Azores, Faial isl. |  | KP981685 |  |  |
| AhFAIT10P15_1 | *A. hamiltoni* | Azores, Faial isl. |  | KP981686 | KP982218 | KP982545 |
| AhFAIT11P2_1 | *A. hamiltoni* | Azores, Faial isl. |  | KP981687 |  |  |
| AhFAIT11P3_1 | *A. hamiltoni* | Azores, Faial isl. |  | KP981688 | KP982219 | KP982546 |
| AhFAIT11P4_1 | *A. hamiltoni* | Azores, Faial isl. |  | KP981689 | KP982220 | KP982547 |
| AhFLOT06P12_1 | *A. hamiltoni* | Azores, Flores isl. |  | KP981690 | KP982221 | KP982548 |
| AhFLOT06P7_1 | *A. hamiltoni* | Azores, Flores isl. |  | KP981691 |  |  |
| AhFLOT06P8_1 | *A. hamiltoni* | Azores, Flores isl. |  | KP981692 |  |  |
| AhFLOT07P1_1 | *A. hamiltoni* | Azores, Flores isl. |  | KP981693 | KP982222 | KP982549 |
| AhFLOT07P9_1 | *A. hamiltoni* | Azores, Flores isl. |  | KP981694 | KP982223 | KP982550 |
| AhFLOT08P3_1 | *A. hamiltoni* | Azores, Flores isl. |  | KP981695 | KP982224 | KP982551 |
| AhFLOT08P6_1 | *A. hamiltoni* | Azores, Flores isl. |  | KP981696 | KP982225 | KP982552 |
| AhPICT01P10_1 | *A. hamiltoni* | Azores, Pico isl. |  | KP981697 | KP982226 | KP982553 |
| AhPICT01P13_1 | *A. hamiltoni* | Azores, Pico isl. |  | KP981698 |  |  |
| AhPICT01P4_1 | *A. hamiltoni* | Azores, Pico isl. |  | KP981699 | KP982227 | KP982554 |
| AhPICT01P6_1 | *A. hamiltoni* | Azores, Pico isl. |  | KP981700 |  |  |
| AhPICT01P9_1 | *A. hamiltoni* | Azores, Pico isl. |  | KP981701 |  |  |
| AhPICT08P7_1 | *A. hamiltoni* | Azores, Pico isl. |  | KP981702 | KP982228 | KP982555 |
| AhPICT09P10_1 | *A. hamiltoni* | Azores, Pico isl. |  | KP981703 | KP982229 | KP982556 |
| AhPICT10P1_1 | *A. hamiltoni* | Azores, Pico isl. |  | KP981704 | KP982230 | KP982557 |
| AhPICT10P11_1 | *A. hamiltoni* | Azores, Pico isl. |  | KP981705 |  |  |
| AhPICT10P15_1 | *A. hamiltoni* | Azores, Pico isl. |  | KP981706 | KP982231 | KP982558 |
| AhPICT10P2_1 | *A. hamiltoni* | Azores, Pico isl. |  | KP981707 |  |  |
| AhPICT10P2_2 | *A. hamiltoni* | Azores, Pico isl. |  | KP981708 |  |  |
| AhPICT10P8_1 | *A. hamiltoni* | Azores, Pico isl. |  | KP981709 |  |  |
| AhPICT10P8_2 | *A. hamiltoni* | Azores, Pico isl. |  | KP981710 |  |  |
| AhSJGT02P14_1 | *A. hamiltoni* | Azores, São Jorge isl. |  | KP981711 |  |  |
| AhSJGT02P2_1 | *A. hamiltoni* | Azores, São Jorge isl. |  | KP981712 |  |  |
| AhSJGT02P5_1 | *A. hamiltoni* | Azores, São Jorge isl. |  | KP981713 |  |  |
| AhSJGT09P10_1 | *A. hamiltoni* | Azores, São Jorge isl. |  | KP981714 |  |  |
| AhSJGT09P5_1 | *A. hamiltoni* | Azores, São Jorge isl. |  | KP981715 |  |  |
| AhSJGT09P8_1 | *A. hamiltoni* | Azores, São Jorge isl. |  | KP981716 |  |  |
| AhSJGT09P9_1 | *A. hamiltoni* | Azores, São Jorge isl. |  | KP981717 |  |  |
| AhSJGT12P5_1 | *A. hamiltoni* | Azores, São Jorge isl. |  | KP981718 | KP982232 | KP982559 |
| AhSMAT01P1_1 | *A. hamiltoni* | Azores, Santa Maria isl. |  | KP981719 |  |  |
| AhSMAT01P12_1 | *A. hamiltoni* | Azores, Santa Maria isl. |  | KP981720 |  |  |
| AhSMAT01P2_1 | *A. hamiltoni* | Azores, Santa Maria isl. |  | KP981721 | KP982233 | KP982560 |
| AhSMAT03P4_1 | *A. hamiltoni* | Azores, Santa Maria isl. |  | KP981722 | KP982234 | KP982561 |
| AhTERT06P2_1 | *A. hamiltoni* | Azores, Terceira isl. |  | KP981723 | KP982235 | KP982562 |
| AhTERT06P3_1 | *A. hamiltoni* | Azores, Terceira isl. |  | KP981724 |  |  |
| AhTERT06P5_1 | *A. hamiltoni* | Azores, Terceira isl. |  | KP981725 | KP982236 | KP982563 |
| AhTERT06P9_1 | *A. hamiltoni* | Azores, Terceira isl. |  | KP981726 | KP982237 | KP982564 |
| AhTERT11P7_1 | *A. hamiltoni* | Azores, Terceira isl. |  | KP981727 | KP982238 | KP982565 |
| AhTERT15P6_1 | *A. hamiltoni* | Azores, Terceira isl. |  | KP981728 |  |  |
| AhTERT15P7_1 | *A. hamiltoni* | Azores, Terceira isl. |  | KP981729 | KP982239 | KP982566 |
| AhTERT18P1_1 | *A. hamiltoni* | Azores, Terceira isl. |  | KP981730 |  |  |
| AhTERT18P4_1 | *A. hamiltoni* | Azores, Terceira isl. |  | KP981731 |  |  |
| AhTERT18P6_1 | *A. hamiltoni* | Azores, Terceira isl. |  | KP981732 | KP982240 | KP982567 |
| AhTERT22P8_1 | *A. hamiltoni* | Azores, Terceira isl. |  | KP981733 | KP982241 | KP982568 |
| AhTERT22P8_2 | *A. hamiltoni* | Azores, Terceira isl. |  | KP981734 | KP982242 | KP982569 |
| AhTERT22P9_1 | *A. hamiltoni* | Azores, Terceira isl. |  | KP981735 | KP982243 | KP982570 |
| AhTERT33P12_1 | *A. hamiltoni* | Azores, Terceira isl. |  | KP981736 |  |  |
| PoFAIT03E3_1 | *P. oromii* | Azores, Faial isl. |  | KP981843 |  |  |
| PoFAIT03E6_1 | *P. oromii* | Azores, Faial isl. |  | KP981844 |  |  |
| PoFAIT03E7_1 | *P. oromii* | Azores, Faial isl. |  | KP981845 |  |  |
| PoFAIT03E7_2 | *P. oromii* | Azores, Faial isl. |  | KP981846 |  |  |
| PoFAIT10I3_1 | *P. oromii* | Azores, Faial isl. |  | KP981847 |  |  |
| PoFAIT10J1_1 | *P. oromii* | Azores, Faial isl. |  | KP981848 | KP982638 | KP982638 |
| PoFAIT10J3_1 | *P. oromii* | Azores, Faial isl. |  | KP981849 | KP982639 | KP982639 |
| PoFAIT10J9_1 | *P. oromii* | Azores, Faial isl. |  | KP981850 |  |  |
| PoFAIT10J9_2 | *P. oromii* | Azores, Faial isl. |  | KP981851 |  |  |
| PoFAIT11E10_1 | *P. oromii* | Azores, Faial isl. |  | KP981852 |  |  |
| PoFAIT11E7_1 | *P. oromii* | Azores, Faial isl. |  | KP981853 |  |  |
| PoFAIT11E7_2 | *P. oromii* | Azores, Faial isl. |  | KP981854 |  |  |
| PoFLOT06J5_1 | *P. oromii* | Azores, Flores isl. |  | KP981855 |  |  |
| PoFLOT06J6_1 | *P. oromii* | Azores, Flores isl. |  | KP981856 | KP982640 | KP982640 |
| PoFLOT06J7_1 | *P. oromii* | Azores, Flores isl. |  | KP981857 | KP982641 | KP982641 |
| PoFLOT06J9_1 | *P. oromii* | Azores, Flores isl. |  | KP981858 |  |  |
| PoFLOT07J1_1 | *P. oromii* | Azores, Flores isl. |  | KP981859 |  |  |
| PoFLOT07J10_1 | *P. oromii* | Azores, Flores isl. |  | KP981860 |  |  |
| PoFLOT07J4_1 | *P. oromii* | Azores, Flores isl. |  | KP981861 | KP982642 | KP982642 |
| PoFLOT07J4_2 | *P. oromii* | Azores, Flores isl. |  | KP981862 | KP982643 | KP982643 |
| PoFLOT07J6_1 | *P. oromii* | Azores, Flores isl. |  | KP981863 | KP982644 | KP982644 |
| PoFLOT08J7_1 | *P. oromii* | Azores, Flores isl. |  | KP981864 |  |  |
| PoFLOT16J1_1 | *P. oromii* | Azores, Flores isl. |  | KP981865 | KP982645 | KP982645 |
| PoFLOT16J10_1 | *P. oromii* | Azores, Flores isl. |  | KP981866 |  |  |
| PoFLOT16J10_2 | *P. oromii* | Azores, Flores isl. |  | KP981867 |  |  |
| PoFLOT16J3_1 | *P. oromii* | Azores, Flores isl. |  | KP981868 | KP982646 | KP982646 |
| PoFLOT16J8_1 | *P. oromii* | Azores, Flores isl. |  | KP981869 | KP982647 | KP982647 |
| PoFLOT16J8_2 | *P. oromii* | Azores, Flores isl. |  | KP981870 | KP982648 | KP982648 |
| PoPICT01I10_1 | *P. oromii* | Azores, Pico isl. |  | KP981871 | KP982649 | KP982649 |
| PoPICT01I4_1 | *P. oromii* | Azores, Pico isl. |  | KP981872 | KP982650 | KP982650 |
| PoPICT01J1_1 | *P. oromii* | Azores, Pico isl. |  | KP981873 | KP982651 | KP982651 |
| PoPICT01L2_1 | *P. oromii* | Azores, Pico isl. |  | KP981874 |  |  |
| PoPICT01L4_1 | *P. oromii* | Azores, Pico isl. |  | KP981875 |  |  |
| PoPICT01L6_1 | *P. oromii* | Azores, Pico isl. |  | KP981876 | KP982652 | KP982652 |
| PoPICT08I1_1 | *P. oromii* | Azores, Pico isl. |  | KP981877 |  |  |
| PoPICT08I7_1 | *P. oromii* | Azores, Pico isl. |  | KP981878 |  |  |
| PoPICT08J3_1 | *P. oromii* | Azores, Pico isl. |  | KP981879 | KP982653 | KP982653 |
| PoPICT09I9_1 | *P. oromii* | Azores, Pico isl. |  | KP981880 | KP982654 | KP982654 |
| PoPICT09J8_1 | *P. oromii* | Azores, Pico isl. |  | KP981881 |  |  |
| PoPICT09J9_1 | *P. oromii* | Azores, Pico isl. |  | KP981882 | KP982655 | KP982655 |
| PoPICT09V9_1 | *P. oromii* | Azores, Pico isl. |  | KP981883 | KP982656 | KP982656 |
| PoSJGT02J3_1 | *P. oromii* | Azores, São Jorge isl. |  | KP981884 |  |  |
| PoSJGT02J6_1 | *P. oromii* | Azores, São Jorge isl. |  | KP981885 |  |  |
| PoSJGT06I3_1 | *P. oromii* | Azores, São Jorge isl. |  | KP981886 |  |  |
| PoSJGT06I4_1 | *P. oromii* | Azores, São Jorge isl. |  | KP981887 |  |  |
| PoSJGT06J2_1 | *P. oromii* | Azores, São Jorge isl. |  | KP981888 |  |  |
| PoSJGT09E2_1 | *P. oromii* | Azores, São Jorge isl. |  | KP981889 |  |  |
| PoSJGT09E5_1 | *P. oromii* | Azores, São Jorge isl. |  | KP981890 |  |  |
| PoSJGT09J8_1 | *P. oromii* | Azores, São Jorge isl. |  | KP981891 |  |  |
| PoSJGT12I6_1 | *P. oromii* | Azores, São Jorge isl. |  | KP981892 |  |  |
| PoSJGT12J5_1 | *P. oromii* | Azores, São Jorge isl. |  | KP981893 | KP982657 | KP982657 |
| PoSJGT12J9_1 | *P. oromii* | Azores, São Jorge isl. |  | KP981894 |  |  |
| PoSMAT01E5_1 | *P. oromii* | Azores, Santa Maria isl. |  | KP981895 |  |  |
| PoSMAT01L5_1 | *P. oromii* | Azores, Santa Maria isl. |  | KP981896 | KP982658 | KP982658 |
| PoSMAT01L8_1 | *P. oromii* | Azores, Santa Maria isl. |  | KP981897 | KP982659 | KP982659 |
| PoSMAT01P10_1 | *P. oromii* | Azores, Santa Maria isl. |  | KP981898 | KP982660 | KP982660 |
| PoSMAT01P6_1 | *P. oromii* | Azores, Santa Maria isl. |  | KP981899 | KP982661 | KP982661 |
| PoSMAT03P3_1 | *P. oromii* | Azores, Santa Maria isl. |  | KP981900 | KP982662 | KP982662 |
| PoSMAT03P9_1 | *P. oromii* | Azores, São Miguel isl. |  | KP981901 |  |  |
| PoSMGGRAM_1 | *P. oromii* | Azores, São Miguel isl. |  | KP981902 |  |  |
| PoSMGT01J9_1 | *P. oromii* | Azores, São Miguel isl. |  | KP981903 |  |  |
| PoSMGTRONQ_1 | *P. oromii* | Azores, São Miguel isl. |  | KP981904 |  |  |
| PoSMGTRONQ_2 | *P. oromii* | Azores, São Miguel isl. |  | KP981905 | KP982663 | KP982663 |
| PoSMGTRONQ_3 | *P. oromii* | Azores, São Miguel isl. |  | KP981906 |  |  |
| PoTERT01J6_1 | *P. oromii* | Azores, Terceira isl. |  | KP981907 |  |  |
| PoTERT01J6_2 | *P. oromii* | Azores, Terceira isl. |  | KP981908 |  |  |
| PoTERT01L6_1 | *P. oromii* | Azores, Terceira isl. |  | KP981909 |  |  |
| PoTERT02J10_1 | *P. oromii* | Azores, Terceira isl. |  | KP981910 |  |  |
| PoTERT02J8_1 | *P. oromii* | Azores, Terceira isl. |  | KP981911 |  |  |
| PoTERT02V7_1 | *P. oromii* | Azores, Terceira isl. |  | KP981912 |  |  |
| PoTERT06J10_1 | *P. oromii* | Azores, Terceira isl. |  | KP981913 | KP982664 | KP982664 |
| PoTERT06J5_1 | *P. oromii* | Azores, Terceira isl. |  | KP981914 |  |  |
| PoTERT06J8_1 | *P. oromii* | Azores, Terceira isl. |  | KP981915 | KP982665 | KP982665 |
| PoTERT11J10_1 | *P. oromii* | Azores, Terceira isl. |  | KP981916 | KP982666 | KP982666 |
| PoTERT11J7_2 | *P. oromii* | Azores, Terceira isl. |  | KP981917 | KP982667 | KP982667 |
| PoTERT11J8_1 | *P. oromii* | Azores, Terceira isl. |  | KP981918 | KP982668 | KP982668 |
| PoTERT15J4_1 | *P. oromii* | Azores, Terceira isl. |  | KP981919 | KP982669 | KP982669 |
| PoTERT15J9_1 | *P. oromii* | Azores, Terceira isl. |  | KP981920 |  |  |
| PoTERT15V3_1 | *P. oromii* | Azores, Terceira isl. |  | KP981921 | KP982670 | KP982670 |
| PoTERT15V3_2 | *P. oromii* | Azores, Terceira isl. |  | KP981922 | KP982671 | KP982671 |
| PoTERT164J5_1 | *P. oromii* | Azores, Terceira isl. |  | KP981923 |  |  |
| PoTERT164L4_1 | *P. oromii* | Azores, Terceira isl. |  | KP981924 | KP982672 | KP982672 |
| PoTERT164L5_1 | *P. oromii* | Azores, Terceira isl. |  | KP981925 |  |  |
| PoTERT173I10_1 | *P. oromii* | Azores, Terceira isl. |  | KP981926 |  |  |
| PoTERT173J9_1 | *P. oromii* | Azores, Terceira isl. |  | KP981927 |  |  |
| PoTERT173L9_1 | *P. oromii* | Azores, Terceira isl. |  | KP981928 |  |  |
| PoTERT18J1_1 | *P. oromii* | Azores, Terceira isl. |  | KP981929 |  |  |
| PoTERT18J7_1 | *P. oromii* | Azores, Terceira isl. |  | KP981930 |  |  |
| PoTERT18L4_1 | *P. oromii* | Azores, Terceira isl. |  | KP981931 |  |  |
| PoTERT22J10_1 | *P. oromii* | Azores, Terceira isl. |  | KP981932 | KP982673 | KP982673 |
| PoTERT22J6_1 | *P. oromii* | Azores, Terceira isl. |  | KP981933 | KP982674 | KP982674 |
| PoTERT22L3_1 | *P. oromii* | Azores, Terceira isl. |  | KP981934 |  |  |
| PoTERT22V5_1 | *P. oromii* | Azores, Terceira isl. |  | KP981935 | KP982675 | KP982675 |
| PoTERT33J7_1 | *P. oromii* | Azores, Terceira isl. |  | KP981936 | KP982676 | KP982676 |
| PoTERT33V4_1 | *P. oromii* | Azores, Terceira isl. |  | KP981937 | KP982677 | KP982677 |
| PoTERT33V8_1 | *P. oromii* | Azores, Terceira isl. |  | KP981938 | KP982678 | KP982678 |
| Ad_FLO_T06_P10_1a | *A. dolosus* | Azores, Flores isl. |  | KP981649 | KP982195 | KP982522 |
| Ad_FLO_T06_P14_1 | *A. dolosus* | Azores, Flores isl. |  | KP981650 |  |  |
| Ad_FLO_T06_Py11_1 | *A. dolosus* | Azores, Flores isl. |  | KP981651 | KP982196 | KP982523 |
| Ad_FLO_T06_Py8_1 | *A. dolosus* | Azores, Flores isl. |  | KP981652 | KP982197 | KP982524 |
| Ad_FLO_T07_Py2_1 | *A. dolosus* | Azores, Flores isl. |  | KP981653 |  |  |
| Ad_PIC_T09_P13_1 | *A. dolosus* | Azores, Pico isl. |  | KP981654 | KP982199 | KP982526 |
| Ad_PIC_T09_P6_1a | *A. dolosus* | Azores, Pico isl. |  | KP981655 | KP982198 | KP982525 |
| Ad_PIC_T09_Py12_1 | *A. dolosus* | Azores, Pico isl. |  | KP981656 |  |  |
| Ad_PIC_T09_Py15_1 | *A. dolosus* | Azores, Pico isl. |  | KP981657 | KP982200 | KP982527 |
| Ad_SMG_T01_P14_1a | *A. dolosus* | Azores, São Miguel isl. |  | KP981658 | KP982201 | KP982528 |
| Ad_SMG_T01_P8_1a | *A. dolosus* | Azores, São Miguel isl. |  | KP981659 | KP982202 | KP982529 |
| Ad_SMG_T01_Py10_1 | *A. dolosus* | Azores, São Miguel isl. |  | KP981660 | KP982203 | KP982530 |
| Ad_SMG_T01_Py3_1 | *A. dolosus* | Azores, São Miguel isl. |  | KP981661 | KP982204 | KP982531 |
| Ad_SMG_T03_P5_1 | *A. dolosus* | Azores, São Miguel isl. |  | KP981662 | KP982205 | KP982532 |
| Ad_SMG_T03_P6_1a | *A. dolosus* | Azores, São Miguel isl. |  | KP981663 |  |  |
| Ad_SMG_T03_Py10_1 | *A. dolosus* | Azores, São Miguel isl. |  | KP981664 | KP982206 | KP982533 |
| Ad_SMG_T03_Py15_1 | *A. dolosus* | Azores, São Miguel isl. |  | KP981665 |  |  |
| Ad_SMG_T04_P5_1 | *A. dolosus* | Azores, São Miguel isl. |  | KP981666 | KP982207 | KP982534 |
| Ad_SMG_T04_Py9_1 | *A. dolosus* | Azores, São Miguel isl. |  | KP981667 |  |  |
| Ad_SMG_T04_Py9_2 | *A. dolosus* | Azores, São Miguel isl. |  | KP981668 | KP982208 | KP982535 |
| Ad_SMG_T07_P8_1 | *A. dolosus* | Azores, São Miguel isl. |  | KP981669 |  |  |
| Ad_SMG_T07_Py10_1a | *A. dolosus* | Azores, São Miguel isl. |  | KP981670 | KP982209 | KP982536 |
| Ad_SMG_T07_Py4_1 | *A. dolosus* | Azores, São Miguel isl. |  | KP981671 | KP982210 | KP982537 |
| Ad_SMG_T07_Py7_1 | *A. dolosus* | Azores, São Miguel isl. |  | KP981672 | KP982211 | KP982538 |
| Ad_TER_T18_P15_1a | *A. dolosus* | Azores, Terceira isl. |  | KP981673 | KP982212 | KP982539 |
| Ad_TER_T18_P6_1a | *A. dolosus* | Azores, Terceira isl. |  | KP981674 |  |  |
| Ad_TER_T18_Py11_1 | *A. dolosus* | Azores, Terceira isl. |  | KP981675 |  |  |
| Ad_TER_T33_P4_1a | *A. dolosus* | Azores, Terceira isl. |  | KP981676 |  |  |
| Ad_TER_T33_P7_1 | *A. dolosus* | Azores, Terceira isl. |  | KP981677 | KP982213 | KP982540 |
| Ad_TER_T33_Py2_1 | *A. dolosus* | Azores, Terceira isl. |  | KP981678 |  |  |

**Table S5.** Pairwise values of genetic differentiation of each studied species between the Azorean islands.

The fixation index Φst (lower matrix) and their corresponding *P* values (upper matrix), assessed by 10,000 permutations and corrected using a sequential Benjamini-Yekutieli procedure, are provided. Significant results (values below 0.05) are in bold face.

| *G. occidentalis* |  | Faial | Flores | Pico | S. Jorge | St. Maria | S. Miguel | Terceira |
| --- | --- | --- | --- | --- | --- | --- | --- | --- |
|  | Faial |  | **0.027** | 0.468 | 0.18 | **<0.001** | **<0.001** | **0.009** |
|  | Flores | 0.294 |  | **0.027** | **<0.001** | **<0.001** | **<0.001** | **0.009** |
|  | Pico | -0.041 | 0.205 |  | 0.198 | **<0.001** | **<0.001** | **0.018** |
|  | S. Jorge | 0.081 | 0.181 | 0.027 |  | **<0.001** | **<0.001** | 0.081 |
|  | St. Maria | 0.795 | 0.832 | 0.784 | 0.721 |  | **<0.001** | **<0.001** |
|  | S. Miguel | 0.908 | 0.96 | 0.905 | 0.911 | 0.425 |  | **<0.001** |
|  | Terceira | 0.171 | 0.174 | 0.155 | 0.107 | 0.5 | 0.526 |  |
| *Sancus acoreensis* |  | Faial | Flores | Pico | S. Jorge | St. Maria | S. Miguel | Terceira |
|  | Faial |  | **<0.001** | 0.603 | 0.25 | **0.007** | **0.015** | 0.596 |
|  | Flores | 0.414 |  | **<0.001** | **<0.001** | **<0.001** | **<0.001** | **<0.001** |
|  | Pico | -0.02 | 0.461 |  | 0.406 | **<0.001** | **<0.001** | **0.018** |
|  | S. Jorge | 0.025 | 0.498 | 0.001 |  | **<0.001** | **0.001** | **0.021** |
|  | St. Maria | 0.204 | 0.497 | 0.173 | 0.225 |  | **<0.001** | **<0.001** |
|  | S. Miguel | 0.118 | 0.461 | 0.097 | 0.103 | 0.216 |  | **<0.001** |
|  | Terceira | -0.014 | 0.383 | 0.024 | 0.04 | 0.091 | 0.099 |  |
| *P. oromii* |  | Faial | Flores | Pico | S. Jorge | St. Maria | S. Miguel | Terceira |
|  | Faial |  | **0.027** | 0.622 | 0.523 | **<0.001** | **0.009** | **<0.001** |
|  | Flores | 0.091 |  | 0.099 | **<0.001** | **<0.001** | **<0.001** | **<0.001** |
|  | Pico | -0.017 | 0.04 |  | 0.486 | **<0.001** | **<0.001** | **<0.001** |
|  | S. Jorge | -0.014 | 0.133 | -0.017 |  | **<0.001** | **0.009** | **<0.001** |
|  | St. Maria | 0.783 | 0.893 | 0.784 | 0.726 |  | **0.027** | **<0.001** |
|  | S. Miguel | 0.843 | 0.957 | 0.843 | 0.791 | 0.272 |  | **<0.001** |
|  | Terceira | 0.45 | 0.517 | 0.435 | 0.345 | 0.729 | 0.755 |  |

**Table S6.** Sequence data variability for each species

| Species | Number of sequences in the analyses | Aligned dataset length in bp | Variable sites | Parsimony informative sites |
| --- | --- | --- | --- | --- |
| *G. occidentalis* | 67 | 1576 | 47 | 28 |
| *Sancus acoreensis* | 94 | 1946 | 191 | 102 |
| *Savigniorrhipis acoreensis* | 76 | 1612 | 234 | 17 |
| *A. hamiltoni* | 30 | 1680 | 145 | 107 |
| *P. oromii* | 41 | 1656 | 101 | 45 |
| *A. dolosus* | 19 | 1495 | 54 | 47 |


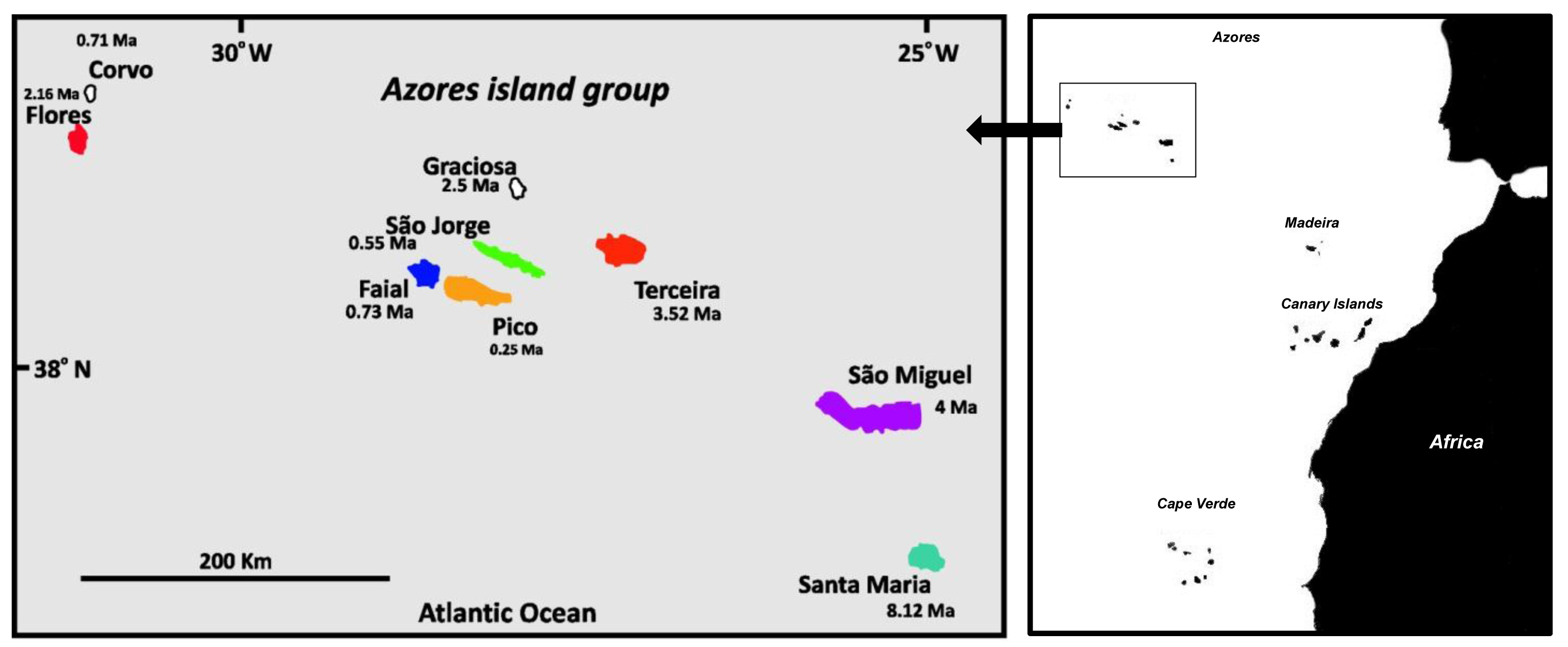


**Figure S1.** Location and map of the Azores. The islands of Corvo and Graciosa were not sampled.

**Figure S2.** Statistical parsimony networks showing relationships among mtDNA cytochrome c oxidase subunit I (COI) haplotypes for (a) *G. occidentalis,* (b), *Sancus acoreensis* and (c) *P. oromii*. Each circle represents a unique haplotype and its size is proportional to its frequency. Branches or black cross-bars represent a single nucleotide change, small black circles represent missing haplotypes, and colours denote collection locations as indicated in the legend.

**References**

1. Borges PAV, Aguiar C, Amaral J, Amorim IR, Andre G, Arraiol A, Baz A, Dinis F, Enghoff H, Gaspar C *et al*: **Ranking protected areas in the Azores using standardised sampling of soil epigean arthropods**. *Biodivers Conserv* 2005, **14**(9):2029-2060.

2. Gaspar C, Borges PAV, Gaston KJ: **Diversity and distribution of arthropods in native forest of the Azores archipelago**. *Arquipélago Life and Marine Sciences* 2008, **25**:1-30.

3. Turquin M-J: **Une biocenose cavernicole originale pour le Bugey: le puits de Rappe. Comptes Rendus 96e Congresse Naturel Sociétès Savantes, Toulouse 1971.** *Sciences* 1973, **3**:235–256.

4. Parmakelis A, Spanos E, Papagiannakis G, Louis C, Mylonas M: **Mitochondrial DNA phylogeny and morphological diversity in the genus *Mastus* (Beck, 1837): a study in a recent (Holocene) island group (Koufonisi, south-east Crete)**. *Biol J Linn Soc* 2003, **78**(3):383-399.
